# Supplementary material for: Protocol for PD SENSORS: Parkinson’s Disease Symptom Evaluation in a Naturalistic Setting producing Outcome measuRes using SPHERE technology. An observational feasibility study of multi-modal multi-sensor technology to measure symptoms and activities of daily living in Parkinson’s disease
Source: BMJ Open. 2020 Nov 30;10(11):e041303. doi: 10.1136/bmjopen-2020-041303 (PMC7705501; doi:10.1136/bmjopen-2020-041303)
Supplement: Supplementary data [file bmjopen-2020-041303supp001.pdf]

Supplementary Material

A list of some of the specific movements/actions to be evaluated in the scripted activities during Contact D

- Spreading hummus on bread
- Cutting
- Pouring
- Scooping
- Stirring
- Turning tap on and off
- Wiping surface
- Dispensing soap

Morgan C, *et al. BMJ Open* 2020; 10:e041303. doi: 10.1136/bmjopen-2020-041303
