# Supplementary material for: Protocol for PD SENSORS: Parkinson’s Disease Symptom Evaluation in a Naturalistic Setting producing Outcome measuRes using SPHERE technology. An observational feasibility study of multi-modal multi-sensor technology to measure symptoms and activities of daily living in Parkinson’s disease
Source: BMJ Open. 2020 Nov 30;10(11):e041303. doi: 10.1136/bmjopen-2020-041303 (PMC7705501; doi:10.1136/bmjopen-2020-041303)
Supplement: Author's manuscript [file bmjopen-2020-041303.draft_revisions.pdf]

BMJ Open

BMJ Open is committed to open peer review. As part of this commitment we make the peer review history of every article we publish publicly available.

When an article is published we post the peer reviewers' comments and the authors' responses online. We also post the versions of the paper that were used during peer review. These are the versions that the peer review comments apply to.

The versions of the paper that follow are the versions that were submitted during the peer review process. They are not the versions of record or the final published versions. They should not be cited or distributed as the published version of this manuscript.

BMJ Open is an open access journal and the full, final, typeset and author-corrected version of record of the manuscript is available on our site with no access controls, subscription charges or pay-per-view fees (http://bmjopen.bmj.com).

If you have any questions on BMJ Open's open peer review process please email info.bmjopen@bmj.com

BMJ Open

# Protocol for PD SENSORS: Parkinson's Disease Symptom Evaluation in a Naturalistic Setting producing Outcomes measuRes using SPHERE technology. An observational feasibility study of multi-modal multi-sensor technology to measure symptoms and activities of daily living in Parkinson's disease.



| Journal: | *BMJ Open* |
|---|---|
| Manuscript ID | bmjopen-2020-041303 |
| Article Type: | Protocol |
| Date Submitted by the Author: | 05-Jun-2020 |





| Complete List of Authors: | Morgan, Catherine; University of Bristol Medical School, Translational Health Sciences; North Bristol NHS Trust, Movement Disorders Group,<br>Craddock, Ian ; University of Bristol, School of Computer Science, Electrical and Electronic Engineering, and Engineering Mathematics, Faculty of Engineering<br>Tonkin, Emma; University of Bristol, School of Computer Science, Electrical and Electronic Engineering, and Engineering Mathematics, Faculty of Engineering<br>Kinnunen, Kirsi; IXICO plc<br>McNaney, Roisin; University of Bristol, School of Computer Science, Electrical and Electronic Engineering, and Engineering Mathematics, Faculty of Engineering<br>Whitehouse, Sam; University of Bristol, School of Computer Science, Electrical and Electronic Engineering, and Engineering Mathematics, Faculty of Engineering<br>Mirmehdi, Majid; University of Bristol, School of Computer Science, Electrical and Electronic Engineering, and Engineering Mathematics, Faculty of Engineering<br>Heidarivincheh, Farnoosh; University of Bristol, School of Computer Science, Electrical and Electronic Engineering, and Engineering Mathematics, Faculty of Engineering<br>McConville, Ryan; University of Bristol, School of Computer Science, Electrical and Electronic Engineering, and Engineering Mathematics, Faculty of Engineering<br>Carey, Julia; University of Bristol, School of Computer Science, Electrical and Electronic Engineering, and Engineering Mathematics, Faculty of Engineering<br>Horne, Alison; University of Bristol Medical School<br>Rolinski, Michal; University of Bristol Medical School, Translational Health Sciences; North Bristol NHS Trust, Movement Disorders Group,<br>Rochester, Lynn; Newcastle University, Institute of Neuroscience; Newcastle Upon Tyne Hospitals NHS Foundation Trust<br>Maetzler, Walter; University Medical Center Schleswig-Holstein Campus Kiel, Department of Neurology<br>Matthews, Helen; Cure Parkinson's Trust |
|---|---|

Watson, Oliver; Bristol Health Partners
Eardley, Rachel; University of Bristol, School of Computer Science, Electrical and Electronic Engineering, and Engineering Mathematics, Faculty of Engineering
Whone, Alan; University of Bristol , School of Translational Health Sciences ; North Bristol NHS Trust, Movement Disorders Group

Keywords: Parkinson-s disease < NEUROLOGY, Information technology < BIOTECHNOLOGY & BIOINFORMATICS, QUALITATIVE RESEARCH, STATISTICS & RESEARCH METHODS

SCHOLARONE™
Manuscripts

*I, the Submitting Author has the right to grant and does grant on behalf of all authors of the Work (as defined in the below author licence), an exclusive licence and/or a non-exclusive licence for contributions from authors who are: i) UK Crown employees; ii) where BMJ has agreed a CC-BY licence shall apply, and/or iii) in accordance with the terms applicable for US Federal Government officers or employees acting as part of their official duties; on a worldwide, perpetual, irrevocable, royalty-free basis to BMJ Publishing Group Ltd ("BMJ") its licensees and where the relevant Journal is co-owned by BMJ to the co-owners of the Journal, to publish the Work in this journal and any other BMJ products and to exploit all rights, as set out in our licence.*

*The Submitting Author accepts and understands that any supply made under these terms is made by BMJ to the Submitting Author unless you are acting as an employee on behalf of your employer or a postgraduate student of an affiliated institution which is paying any applicable article publishing charge ("APC") for Open Access articles. Where the Submitting Author wishes to make the Work available on an Open Access basis (and intends to pay the relevant APC), the terms of reuse of such Open Access shall be governed by a Creative Commons licence – details of these licences and which Creative Commons licence will apply to this Work are set out in our licence referred to above.*

*Other than as permitted in any relevant BMJ Author's Self Archiving Policies, I confirm this Work has not been accepted for publication elsewhere, is not being considered for publication elsewhere and does not duplicate material already published. I confirm all authors consent to publication of this Work and authorise the granting of this licence.*

For peer review only

# Protocol for PD SENSORS: Parkinson's Disease Symptom Evaluation in a Naturalistic Setting producing Outcomes measuRes using SPHERE technology. An observational feasibility study of multi-modal multi-sensor technology to measure symptoms and activities of daily living in Parkinson's disease.

Authors: Catherine Morgan [a,c,*] , Ian Craddock [b], Emma Tonkin [b], Kirsi M Kinnunen [d], Roisin McNaney [b], Sam Whitehouse [b], Majid Mirmehdi [b], Farnoosh Heidarivincheh [b], Ryan McConville [b], Julia Carey [b], Alison Horne [e], Michal Rolinski [a,c], Lynn Rochester [f,g], Walter Maetzler [h], Helen Matthews [i], Oliver Watson [j], Rachel Eardley [b], Alan L Whone [a,c]

[a] University of Bristol Medical School, Translational Health Sciences, Bristol, UK

[b] University of Bristol, School of Computer Science, Electrical and Electronic Engineering, and Engineering Mathematics, Faculty of Engineering, Bristol, UK

[c] North Bristol NHS Trust, Movement Disorders Group, Westbury on Trym, Avon, UK

[d] IXICO plc, London, UK

[e] University of Bristol Medical School, Bristol, UK

[f] Newcastle University, Institute of Neuroscience, Newcastle, UK

[g] Newcastle Upon Tyne Hospitals NHS Foundation Trust, Newcastle Upon Tyne, UK

[h] University Medical Center Schleswig-Holstein Campus Kiel, Department of Neurology, Kiel, Schleswig-Holstein, DE

[i] Cure Parkinson's Trust, London, UK

[j] Bristol Health Partners, Bristol, UK

**Emails of authors**: Catherine.morgan@bristol.ac.uk; ian.craddock@bristol.ac.uk; e.l.tonkin@bristol.ac.uk; kirsi.kinnunen@ixico.com; r.mcnaney@bristol.ac.uk; sw12690@bristol.ac.uk; m.mirmehdi@bristol.ac.uk; farnoosh.heidarivincheh@bristol.ac.uk; ryan.mcconville@bristol.ac.uk; Julia.carey@bristol.ac.uk; alison.horne@bristol.ac.uk; michal.rolinski@bristol.ac.uk; lynn.rochester@newcastle.ac.uk; w.maetzler@neurologie.uni-kiel.de; helen@cureparkinsons.org.uk; oliver.watson@bristol.ac.uk; Rachel.eardley@bristol.ac.uk; alan.whone@bristol.ac.uk

**Correspondence to:** Dr Catherine Morgan; Catherine.morgan@bristol.ac.uk; Movement Disorders Group, Bristol Brain Centre, Southmead Hospital, North Bristol National Health Service Trust, Bristol, UK; +44 7970 657958

**Registration:** University of Bristol Research Registration Database, reference 2018-4247



For peer review only - http://bmjopen.bmj.com/site/about/guidelines.xhtml

UOB Confidential

For peer review only

**Keywords:** Parkinson's disease; Information technology; Qualitative research; Statistics and research methods

**Word count:** 4702

**Abstract**

Introduction: The impact of disease-modifying agents on disease progression in Parkinson's disease is largely assessed in clinical trials using clinical rating scales. These scales have drawbacks in terms of their ability to capture the fluctuating nature of symptoms while living in a naturalistic environment. The SPHERE (Sensor Platform for HEalthcare in a Residential Environment) project has designed a multi-sensor platform with multimodal devices designed to allow continuous, relatively inexpensive, unobtrusive sensing of motor, non-motor and activities of daily living metrics in a home or a home-like environment. The aim of this study is to evaluate how the SPHERE technology can measure aspects of Parkinson's disease.

Methods and Analysis: This is a small-scale feasibility and acceptability study using a cohorts of people with Parkinson's disease and healthy volunteer control participants. Analytic work-up of a number of diverse outcomes in Parkinson's will be undertaken, including looking at bradykinesia, urinary dysfunction and food preparation tasks. Acceptability of the technology will be evaluated qualitatively using semi-structured interviews.

Ethics and Dissemination: Ethical approval has been given to commence this study; the results will be disseminated as widely as appropriate.

**Article summary**

This feasibility pilot study looks to explore the how best to use the SPHERE technology to measure aspects of PD, and which symptoms or activities are possible and useful to evaluate.

Strengths and limitations of this study:

- The explorative/iterative design will allow flexibility in the analytic work-up which will then be used to inform a future longer-term study utilising the learning from PD SENSORS.
- The multimodal multisensor platform design allows to chance to evaluate multiple symptoms and activities in an unobtrusive, continuous and relatively inexpensive way in the participants' home or home-like environment.
- The small number of participants means that results generated from this study will not be generalizable to the wider population of people with PD.
- Due to the choice to include only those with mild to moderate PD, the data will not therefore be generalizable to people with more severe disease.
- The feasibility nature of PD SENSORS means that cost effectiveness will not be explored at this stage.



For peer review only

For peer review only - http://bmjopen.bmj.com/site/about/guidelines.xhtml

UOB Confidential

## Introduction

Parkinson's disease (PD) is the second most common neurodegenerative disease in the UK, affecting more than 127,000 people. It is a chronic, progressive, disabling disease which is characterised by a large variety of possible motor symptoms (including bradykinesia, rigidity and tremor) and non-motor symptoms (including sleep disturbance, cognitive impairment and pain) (1). It is estimated that by the time of diagnosis with PD most patients have already lost around 50% of their dopaminergic neurones (2), therefore attempts to modify the disease should be aimed at pre-clinical or early-stage disease states.

The search for a treatment which could modify PD, a so-called disease-modifying therapy or 'DMT', has thus far proven fruitless with no DMT reaching market authorisation. This is despite multiple potential therapies having been tested in randomised, double-blind clinical trials (3).

Clinical trials testing DMTs have often used a clinical rating scale to quantify symptoms and monitor response to treatment as a primary outcome measure (3). The gold-standard rating scale is the Movement Disorder Society-sponsored revision of the Unified Parkinson's Disease Rating Scale (MDS-UPDRS) (4). This is a validated (5) tool which evaluates a comprehensive spectrum of symptoms and activities. However it is also a subjective (6) and non-linear (7) tool which exposes the study participant to the risk of observer bias and the 'Hawthorne Effect' (8). It is used episodically, giving a snapshot of a person with PD, which may not capture the day-to-day and hour-to-hour fluctuations of this complex disease. The testing location is often an artificial environment like a clinic or laboratory. This can increase costs (9) and impair the ability of studies to appraise important activities of daily living (ADLs) such as engagement in social activities (10), rare events such as falls (11) and other metrics which affect wellbeing and quality of life such as sleep (12).

Development of technologies for the measurement of various aspects of PD have been evolving over the past 15 years (13). Particularly motor symptoms, including gait and bradykinesia, are amenable to measurement by technology (14) continuously and over long time periods. For example, the Parkinson's KinetiGraph wearable movement recording device (developed by Global Kinetics) can measure tremor, bradykinesia and dyskinesia(15) and the Microsoft Kinect motion sensor can classify PD stages relating to gait impairment (16). However, these tools are still only partially integrated into clinical trials, with most continuing to use periodic clinical rating scale assessments in the non-ecological setting as primary or secondary end-points (17). There are excellent initiatives which are guiding this field forward, for example a 'roadmap' for implementation of digital outcome measures using technologies (18), which are tackling the important questions surrounding regulatory approvals and collaboration with industry.

Lacunae in the literature about technology-assisted outcomes in PD include the measurement of non-motor outcomes (14) such as constipation and activities of daily living. The frequent use of wearable devices which are attached to specific body parts means that the information gathered is restricted to the movement in that part of the body; this may be inadequate fully to understand the context in which a measurement is taken and may result in low correlations with measures of functional disability and/or quality of life (19). In addition, it can be challenging to establish and operationalise the 'ground truth' for benchmarking novel measurements. Reviews of technology-assisted outcome measures in a free-living environment have highlighted that to date there is no



For peer review only - http://bmjopen.bmj.com/site/about/guidelines.xhtml

UOB Confidential

For peer review only

fully-validated system which evaluates clinical features of PD in a naturalistic/ecologically valid (i.e. home or home-like) environment (20).

**Sensor Platform for HEalthcare in a Residential Environment - SPHERE**

The long-standing SPHERE project from the University of Bristol (SPHERE: Sensor Platform for HEalthcare in a Residential Environment) has led to the design of a multi-sensor platform with multimodal devices designed to allow continuous, relatively inexpensive, unobtrusive sensing of motor, non-motor and ADL metrics in a home or a home-like environment (21,22).

Within the SPHERE house there are a number of sensors which include: wearable devices with accelerometers (in-house developed); environmental/ambient sensors which measure temperature, humidity and light levels; RGB-D ('red green blue – depth', referring to the three colour planes that together can generate the colour of a pixel in an image, in addition to the recording of distances to surfaces using infra-red light) camera sensors that generate silhouettes of participants and bounding boxes that, when linked with the SPHERE wearable, let the system know who the participant is; appliance sensors which are able to tell when and how frequently an appliance is switched on and off; PIR (Passive InfraRed) sensors which detect motion in each room.

The SPHERE sensor platform has undergone extensive testing with healthy volunteers, being deployed to 50 homes over a year from January 2017. High-quality qualitative work has been conducted looking at the design and acceptability of the SPHERE sensors and technology in a person's life (23,24). In addition, the SPHERE platform is being utilised in 3 clinical projects which are at different stages of completion, looking at outcomes around cardiac valve replacement, hip and knee surgery (25) and in dementia/mild cognitive impairment.

The multimodal multi-sensor design of the SPHERE system (22) enables a richer and more fine-grained picture of free-living symptoms and activities to be collected. For example, the combined use of the wearable devices with accelerometers, alongside the video and environmental sensors, enables machine learning algorithms to opportunistically and continuously fuse data to build a time series of activities of daily living for each person in the home (26,27)This offers an exciting opportunity to conduct free-living evaluation of people with PD.

**Parkinson's Disease Symptom Evaluation in a Naturalistic Setting producing Outcomes measuRes using SPHERE technology – PD SENSORS**

Given the above gaps in knowledge, and appreciating the SPHERE system's capability, it is believed that SPHERE technology could be exploited for filling some of the gaps in the current use of technology to measure outcomes in PD (e.g. for non-motor symptoms and ADLs). PD SENSORS is a feasibility and acceptability study using the SPHERE platform to develop novel outcome measures in PD. It is designed with the intent that it will inform a future larger scale and longer-term investigation, in which SPHERE technology will be deployed to multiple homes of people with PD (protocol version 1.2; May 2020). This is a truly collaborative study with not only strong interdisciplinary collaboration between computer scientists and movement disorders clinicians but also industry involvement. The university academic team are working with a UK-based imaging and digital biomarker analysis company, IXICO, that contributes clinical and data science expertise and is



For peer review only - http://bmjopen.bmj.com/site/about/guidelines.xhtml
UOB Confidential

providing additional wearable devices from Axivity (AX3) and Activinsights (GENEActiv Original) and gait mat technology from ProtoKinetics (Zeno instrumented walkway), to further enrich the dataset.

## Aims

### Primary aims

To understand how the raw sensor data obtained from the SPHERE system can be processed to translate into meaningful data for the clinical scientist on metrics of:

- Total body movement
- Bradykinesia
- Sleep quantity and quality
- Urinary dysfunction
- Meal preparation/consumption
- Cleaning tasks
- Getting out of a chair
- Activity level (ambulatory activity and sedentary behaviour)
- Room to room transfers and time spent in each room

### Secondary aims

1. To evaluate the acceptability of the continuous use of multiple and varied SPHERE sensors over a 5-day period in a home-like setting for people with Parkinson's disease and to explore the participants' experience of such a study
2. To explore the use of home sensing as a means to discriminate between participants with PD and healthy control participants
3. To assess the criterion validity of the technology-assisted outcomes by mapping the sensor data outcomes to the gold-standard clinical rating scale (MDS-UPDRS) and patient-reported outcome measure (PDQ-39) when participants with PD are in both 'on' and 'off' medication states

## Methods

### Design

A small-scale feasibility and acceptability study.

### Setting and participants

The setting will be the SPHERE house: a customised 2-bedroom residential house in Bristol, UK, with multiple embedded sensors and data collection tools.

12 pairs of participants comprising a person with PD and an age-matched control participant (likely to be a spouse, other family member, friend, or carer) will be recruited to stay in the SPHERE house for 5 consecutive days.

Participants with PD will be eligible to enter the study if they meet certain inclusion criteria, including:



For peer review only



UOB Confidential

- Diagnosis of idiopathic Parkinson's disease according to UK Brain Bank Criteria(28);
- Age over 18
- Modified Hoehn and Yahr Scale score of 3 or less in 'off' state (i.e. when they withhold dopaminergic medications)

Inclusion criteria for control participants includes:

- No history of Parkinson's disease, REM (rapid eye movement) sleep behaviour disorder, dementia, or other neurodegenerative/significant musculoskeletal condition

The study's exclusion criteria include:

- Montreal Cognitive Assessment Score < 26
- Current active depressive symptoms; Beck Depression Inventory II score >19
- Use of walking aids whilst inside a house to aid mobility either 'on' or 'off' medications

Exclusion criteria were largely chosen to reduce the potential impact on sensor data of other factors, such as mobility aids altering accelerometry data making it difficult to evaluate bradykinesia.

Participants will be recruited through movement disorders specialist or general neurology outpatient clinics in North Bristol NHS Trust and through posters in the outpatient department of North Bristol NHS Trust, The Cure Parkinson's Trust, a local Patient and Public Involvement Group or by word-of-mouth.

The medical PD care for those study participants with the disease will continue unchanged.

Full approval from the National Health Service (NHS) Wales Research Ethics Committee 6 was granted on 17th December 2019, and Health Research Authority and Health and Care Research Wales approval confirmed on 14th January 2020.

**Overview of study contacts**

PD SENSORS is split into 5 study 'contacts' (stages at which information is transmitted to or gathered from the study participants). A flow chart in Figure 1, below, illustrates the main aims of each study contact.

At each study contact, specific information is gathered from the participants by the research team. Table 1 details the tools to be used during the longer study contacts, Contacts B and D, with the participant with PD. The items in italics in this table apply only to the participant with PD and not to the control participant.

Table 1. Baseline and follow-up data from Contacts B and D

| Time Point | Outcomes Measured |
|---|---|
| **Contact B** | Further demographic information including date of birth and *date of diagnosis of PD*<br>Full screening questions according to inclusion and exclusion criteria<br>*Medication regime and calculation of Levodopa equivalent daily dose*<br>*H&Y score*<br>*MOCA* |



For peer review only - http://bmjopen.bmj.com/site/about/guidelines.xhtml

UOB Confidential

For peer review only

| | |
|---|---|
| | *BDI II, AS, ESS*<br>*Timed Up and Go Test*<br>*NMSS*<br>*PDSS*<br>*RBD-SQ* |
| **Contact D (the SPHERE house stay)** | *MDS-UPDRS (the motor sub-scale will be performed on multiple occasions, once while 'off' medications)*<br>*PDQ-39*<br>Symptom and activity diary<br>Sleep diary<br>Bladder diary<br>*Medication-taking record*<br>Sensor data from scripted activities<br>Sensor data from free-living<br>Sensor data from wearable devices provided through IXICO<br>Annotations of colour video dataset<br>Gait mat assessments<br>Interview with questions relating to experience of living with SPHERE technology |

*AS = Apathy Scale; BDI II = Beck's Depression Inventory II; ESS = Epworth Sleepiness Scale; H&Y = Hoehn and Yahr; MDS-UPDRS = Movement Disorder Society sponsored revision of the Unified Parkinson's Disease Rating Scale; MOCA = Montreal Cognitive Assessment; NMSS = Non-Motor Symptoms Scale for Parkinson's disease; PDQ-39 = Parkinson's Disease Questionnaire-39; PDSS-2 = Parkinson's Disease Sleep Scale-2; RBD-SQ = REM sleep Behaviour Disorder Screening Questionnaire*

For peer review only

**Consent**

Informed consent will be sought from the potential participants by the Principal Investigator, CM, at Contact B after distributing the study Participant Information Leaflet in advance and giving opportunity to discuss its contents and answer any questions.

**Schedule of contact D: the stay in the SPHERE house**

The pair of participants will stay in the house together for five days. The duration of stay was chosen in consultation with people with PD and their acquaintances who took part in pre-study focus groups. This stay in the SPHERE house for each pair of participants will involve:

- Brief specific scripted activities relevant to our planned outcome measurements during the SPHERE house stay, witnessed and annotated by a study team member during their planned visits to the house. These activities will analyse food preparation and cleaning/hygiene activities (more detail about these activities is shown in the supplementary material).
- The participant with PD withholding their dopaminergic medications for >12 hours so that they are practically-defined 'off' medications. During this time in the 'off' medication state, some assessments will be undertaken including the MDS-UPDRS motor subsection. These assessments will then be repeated after the participant with PD restarts their medications.
- Additional wearable devices provided by IXICO: one wearable will be worn on each wrist throughout the SPHERE house stay and for a short period of time on day 4 one additional wearable will be placed around the trunk so that the sensor is in the lower back (lumbar) area and another one on a lower limb (shin) to the study to compare algorithm and sensor data collection.



For peer review only - http://bmjopen.bmj.com/site/about/guidelines.xhtml

UOB Confidential

Figure 2 shows a day-by-day plan for Contact D for the pair of participants.

Gait mat assessments

During the 'off' and 'on' medication testing session on Day 4, the participants will undertake clinical assessments including an evaluation of gait on the Zeno Walkway, a gait mat embedded with sensors that is placed in the entrance area of SPHERE house where these assessments are conducted. The item is administered according to the standard instructions and the gait mat protocol developed by ProtoKinetics. All gait mat protocols for these assessments have been developed by ProtoKinetics, the Zeno walkway manufacturers.

Colour video capture

In communal areas of the SPHERE house (i.e. not in bedrooms or bathroom) cameras will be enabled to produce RGB data for the purposes of allowing more accurate and relevant evaluation of the participants' movements. Short periods of these data will be used in the analytic work-up of total body movement using annotations of this dataset relating to the participants' activities, location, and aspects of their Parkinson's disease symptomatology.

All work during the events and circumstances relating to severe acute respiratory syndrome coronavirus (SARS-COV-2) or Coronavirus-19 (COVID-19) will be subject to risk assessment and modification to ensure the study is safe for participants and researchers.

**Additional outcomes**

The University of Bristol and IXICO will collaborate on specific outcomes, as well as through the sharing of data and results, the loan of the wearable biosensors (Axivity AX3 and GENEActiv Original) and gait mat (Zeno walkway) and in-kind contributions of scientific expertise and study operations. The topics explored within this collaboration will include the feasibility of labelling total body bradykinesia from colour video, to developed automated detection of bradykinesia from biosensor data, biosensor device comparability, and relationships between sensor-based measurements from the gait mat and wearable devices and between sensor-based measurements and traditional clinical measures of motor symptoms, and ADL detection from wearable sensor data.

**Qualitative work**

A semi-structured interview will be conducted with each participant at the end of the study exploring their experience of the SPHERE technology, with a focus on the tolerability and acceptability of such technology for people with PD and those close to them. An interview topic guide is shown in Box 1. Interview transcripts will be read, re-read, and the data gathered will be analysed thematically, using an inductive approach, with methods drawn from Braun and Clarke (29).

For peer review only



UOB Confidential

Box 1. Interview Topic Guide

- Background experience with technology
- Experience of the SPHERE sensor platform
- Thoughts about the sensor data collected
- The study information received relating to the SPHERE technology
- Questions about the study experience specific to PD and those close to people with PD

Additionally, two questionnaires will be used to augment the qualitative data produced from the interviews. The first questionnaire will be a standardised tool evaluating the participants' attitudes towards and experience of technology: using four attitude-based subscales (looking at positive attitudes, negative attitudes, technological anxiety/dependence, and attitudes towards task-switching) from The Media and Technology Usage and Attitudes Scale (30). This will allow us to interpret the interview responses in the context of a participant's prior background exposure to technology. The second questionnaire comprises three short questions exploring feedback about the sensors or study in general regarding thoughts that could have occurred to the participant in the days after they left the SPHERE house. These questions are listed in Box 2. This enables us to ensure we understand the experience of this sensor platform as fully as possible.

Box 2. Questions following-up the SPHERE house stay (Contact E questionnaire)

1. Following the PD SENSORS study, now that you have finished the study and been at home for a while, do you have any further thoughts to feedback to us about the SPHERE sensors that you lived with for 5 days?
2. Specifically, in relation to your PD/supporting role for a person with PD, was there anything further you wanted us to know about or understand?
3. Is there anything else at all that you felt important to mention to us regarding your study experience?

**Safety reporting**

Any adverse event (AE) will be assessed and acted upon by the research team. All AEs will be recorded in the study or project file with a note that will identify when the event occurred, the details of the AE, any potential study relation, action taken and resolution / closure of the AE. An assessment of seriousness will be made by the researcher and serious adverse events (SAEs) will be reported in line with current legislation and university guidance.

In addition, all SAEs will be reported to the NHS Research Ethics Committee in the Annual Progress Report.

**Amendments**

Any amendments to protocol design will be communicated to relevant parties including North Bristol NHS Trust and the Research Ethics Committee.

**Sample size**



For peer review only



UOB Confidential

This sample size (24) was chosen as a balance between gathering enough data from the SPHERE sensors to create excellent quality algorithms (in consultation with our Machine Learning team) and the need to pragmatically take into account the nature of this exploratory study which is developing analytics to use in deployment to people's own homes in a future study. No conventional sample size calculation has been undertaken since we have no clear effect estimates to use in any such calculation; we view this more as a pilot and a proof of concept study. The comparator of the healthy volunteer control participant was chosen in order to make the study more sociable and therefore hopefully enjoyable to the person with PD, alongside also providing study data from people without PD so that conclusions could be drawn about how to identify and then measure aspects of PD.

**Statistical analysis**

Study researchers and the study statistician will work collaboratively with the clinicians and SPHERE machine learning team to determine how the SPHERE data can be transformed into usable form. The research team will then aim to ascertain if this data can give us a signal to discriminate between participants with PD and control participants and whether it can give us a signal to discriminate between when a participant with PD is 'on' or 'off' medications. We will compare it to the data produced by the gold-standard clinical rating scales and patient-reported outcome measures. Machine learning (ML) will play a key role in analysing the data.

Existing works have proposed methods based on ML for PD diagnosis and monitoring (please refer to [34] for a detailed review). Using various types of data, such as videos, Inertial Measurement Unit (IMU) and smart-phone or wearable accelerometer data, they model the presence and/or severity of PD via ML algorithms such as Artificial Neural Networks (ANN), Hidden Markov Models (HMM), Support Vector Machines (SVM) and Random Forests (RF). We also aim to use the data collected from the SPHERE house, which is a multi-sensor platform, with various ML techniques to assess the reliability of an automatic learning approach for modelling and analysing PD. As mentioned before, the data that will be utilised to learn from includes the output of wearable accelerometer, RGB-D cameras with silhouette extraction, PIR and environmental sensors.

To achieve such analysis, multiple potential approaches could be considered for future work, some of which are presented in the following:

**PD vs non-PD recognition –** Given the input data corresponding to different time scales, such as seconds, minutes, or hours, can an ML-based model be trained to discriminate between PD and non-PD (control) subjects. Such recognition could be performed as binary classification or as a probability estimation per category. Further, the predictions corresponding to different durations could be compared to determine the one by which the most reliable recognition is achieved.

**PD severity estimation –** Based on different rating scales such as UPDRS or *H&Y* score, an ML-based approach could learn to estimate the severity of the disease among the PD patients. The ground-truth scores are provided by the clinicians during both 'on' and 'off' medication periods. Then, a model could be trained to regress predictions using the ground-truth scores.



For peer review only - http://bmjopen.bmj.com/site/about/guidelines.xhtml

UOB Confidential

For peer review only

**Fine-grained analysis of PD symptoms –** In addition to PD recognition and severity estimation, an ML-based model could be trained to detect different PD symptoms, such as bradykinesia or tremor. Also, a finer-grained analysis of each symptom could be investigated. For example, a model could learn to detect peaks or changes during tremor.

**Activity recognition –** The learning could be extended towards recognising the category of the activities which are performed by the subjects during free living or scripted activity experiments. Then, the quality of the performed activities such as meal preparation or cleaning, could be assessed. Also, the relation between PD symptoms like bradykinesia, and the performed activities could be modelled

**Behaviour analysis of the PD patients –** ML algorithms could be applied to model the behavioural patterns of PD vs non-PD subjects. Such models could be also trained to distinguish between the behaviour of the PD patients during 'on' and 'off' medication periods. Moreover, the patterns in the sleeping habits, mobility, and urinary function of the patients could be learned and analysed.

**Modelling the progression of PD temporally –** The above-mentioned predictions could be monitored through time by modelling the progression of the disease via ML algorithms that provide temporal analysis of the input data. Specifically, models like HMMs or Recurrent Neural Networks (RNNs), which can produce predictions per time point, could be trained to temporally model the PD symptoms. Therefore, for example, PD symptoms could be assessed at different times of a day during the course of the study.

**Analysing the information collected from screening and diaries –** The questionnaires and tests performed during screening (Contact B), as well as the diaries filled by the patients during their stay in the SPHERE house, would provide information which could be summarised via descriptive statistics. Additionally, associations with the data collected from various sensors, for example, environmental sensors, could be modelled via ML.

**Evaluating traditional vs state-of-the-art ML algorithms –** As presented in [34], there are many ML algorithms which can be applied to model PD. We would have the ability to assess the performance of such alternative algorithms. Specifically, a comparison between classical ML algorithms, such as SVMs or RFs, against state-of the-art deep learning-based approaches for modelling PD is of interest, as the latter has achieved high performance in areas such as computer vision.

**Evaluating single vs multi-modal approaches –** Due to the multi-sensor data collection platform within the SPHERE house, the performance of ML models which are based on single or multiple modalities could be assessed. In a multi-modal approach, methods for fusing data from different modalities, e.g. early or late fusion methods, could be also evaluated to determine which modalities are most effective.

**Data management and security**

The day-to-day management of the study including data management will be coordinated by the Principal Investigator of the study. All the data collected will be fully anonymised or pseudonymised where possible. Unique study identification numbers will be assigned to each participant. Access to data will be limited to SPHERE researchers and collaborators only, who must be registered (to use



For peer review only - http://bmjopen.bmj.com/site/about/guidelines.xhtml

UOB Confidential

the data) with managed access and permissions. Directly-identifiable data (e.g. name, address, telephone number) will be held securely and separately from other study data. Data procedures will be in accordance with the General Data Protection Regulation. If a participant withdraws, the data that is already obtained will be kept by the study team. A Data Monitoring Committee is not needed because of the feasibility testing nature of this work, however under a Service Level Agreement between the University of Bristol and University Hospitals Bristol NHS Foundation Trust, the Trust will monitor 10% of the University's Sponsored studies. In line with the National Institute for Health and Research guidance which encourages sharing anonymised datasets, we will seek informed consent from participants for their anonymous data to be shared with other researchers. The data management will be reviewed on a regular basis by an in-house Data Monitoring Committee consisting of data experts, engineers and movement disorders clinicians.

**Patient and public involvement**

The Bristol Movement Disorders Health Integration Team (MOVE-HIT) has an active patient and public involvement (PPI) group which includes people with Parkinson's disease and their partners/carers. The MOVE-HIT PPI group meets regularly and has been involved in providing extensive advice around the study design, as well as the development of the participant information sheets, consent forms and participant diaries. The opinions of members of this PPI group on study design have been collected both in face-to-face group and individual consultations and in a survey to wider group members. We plan further ongoing consultation, both during the middle of the study to discuss the group's views on study progress and at the end of the study to consult on the plain English report on the study findings and to identify dissemination outlets.

The PD SENSORS research team are also collaborating with Lyndsey Isaacs, a Trustee of The Cure Parkinson's Trust (CPT), who facilitated the presentation of a PD SENSORS study design survey to the delegates at a CPT Research Update Meeting. This survey was also made more widely available on the CPT website. The research team will have ongoing liaison with CPT representatives during the study to discuss aspects of study management such as dissemination of research findings. The CPT have agreed to be a forum through which to disseminate the study Poster to allow interested persons to express interest in participating in PD SENSORS.

**Ethics and Dissemination**

The SPHERE house has been safety checked by an experienced occupational therapist practitioner with an interest in PD and relevant adaptations made.

It is anticipated that the scientific results of the study and its various elements will be published both via presentation at national or international conferences and through publication in peer-reviewed scientific journals/publications.

**Data Statement**

SPHERE intends to make any research data produced as widely and freely available as possible in a timely and responsible manner. The information would be suitable for sharing as we are developing novel and potentially useful ways to assess PD. However, some portions of research data will not be suitable for sharing, e.g., directly identifiable information such as names and addresses. We will seek consent from participants for pseudonymised subsets of their data to be shared with other



For peer review only

For peer review only - http://bmjopen.bmj.com/site/about/guidelines.xhtml

UOB Confidential

researchers. This is in anticipation that data sharing and access to such datasets may become mandatory in the coming years.

Data published by the University of Bristol (UoB) Repository can have open or controlled data access. Published datasets will appear in a standardised, online 'data catalogue' accompanied by its deposit record. These deposits are also made visible to search engines such as Google, to help maximise their visibility and impact. We will also create records of digital and/or non-digital data produced in Pure (http://www.bristol.ac.uk/red/research-policy/pure/). Our approach to data sharing will be published on the Digital Health website which is allied to the University of Bristol Engineering website and is accessible to everyone from researchers to the wider general public.

**Funding**

This work was performed under the SPHERE Next Steps Project funded by the UK Engineering and Physical Sciences Research Council (EPSRC), Grant EP/R005273/1.

This work is supported by the Elizabeth Blackwell Institute for Health Research, University of Bristol, and the Wellcome Trust Institutional Strategic Support Fund, grant code: 204813/Z/16/Z, and by The Cure Parkinson's Trust, grant code AW021.

Dr Jonathan de Pass and Mrs Georgina de Pass made a charitable donation to the University of Bristol (of which Jonathan is a graduate) through the Development and Alumni Relations Office to support research into Parkinson's Disease. The funding pays for the salary of CM, the PhD student and Clinical Research Fellow, and she reports to the donors on her progress, but they do not have any influence on the direction of the study.

**Author Statement**

CM drafted the study protocol, worked with all contributors to improve and finalise details and continues as the study Principal Investigator. IC and AW supervise the studies and work of CM; AW is study Chief Investigator, IC is Principal of SPHERE-IRC; both have major influence on the study's direction. ET has advised on data management. KK and RM have done pilot work and have advised on study direction. MM, FH, RC, ET and SW have been involved in the computer science and analytics planning. JC, AH, MR and RE have given practical input on study tools and documentation. OW and HM are representatives of the Public and Patient Involvement groups which have had strong influence on study design. LR and WM have added expertise in the field to advise CM on early study design.

The study sponsor is the University of Bristol (research-governance@bristol.ac.uk). The sponsor and funders have had no role in study design, collection, management, analysis, interpretation of the data, writing the report or the decision to submit the study for publications and will have no ultimate authority over these activities.

**Conflicts of interests**

No financial or other competing interests to declare.

**References**



For peer review only

For peer review only - http://bmjopen.bmj.com/site/about/guidelines.xhtml

UOB Confidential

1. Jankovic J. Parkinson's disease: clinical features and diagnosis. J Neurol Neurosurg Psychiatry [Internet]. 2008 Apr 1 [cited 2018 Nov 15];79(4):368–76. Available from: http://www.ncbi.nlm.nih.gov/pubmed/18344392

2. Fearnley JM, Lees AJ. Ageing and Parkinson's disease: substantia nigra regional selectivity. Brain [Internet]. 1991 Oct [cited 2018 Nov 15];114 ( Pt 5):2283–301. Available from: http://www.ncbi.nlm.nih.gov/pubmed/1933245

3. Lang AE, Melamed E, Poewe W, Rascol O. Trial designs used to study neuroprotective therapy in Parkinson's disease. Mov Disord. 2013;28(1):86–95.

4. Goetz CG, Fahn S, Martinez-Martin P, Poewe W, Sampaio C, Stebbins GT, et al. Movement Disorder Society-sponsored revision of the Unified Parkinson's Disease Rating Scale (MDS-UPDRS): Process, format, and clinimetric testing plan. Mov Disord [Internet]. 2007 Jan [cited 2020 Feb 3];22(1):41–7. Available from: http://doi.wiley.com/10.1002/mds.21198

5. Goetz CG, Tilley BC, Shaftman SR, Stebbins GT, Fahn S, Martinez-Martin P, et al. Movement Disorder Society-Sponsored Revision of the Unified Parkinson's Disease Rating Scale (MDS-UPDRS): Scale presentation and clinimetric testing results. Mov Disord. 2008 Nov 15;23(15):2129–70.

6. Post B, Merkus MP, de Bie RMA, de Haan RJ, Speelman JD. Unified Parkinson's disease rating scale motor examination: are ratings of nurses, residents in neurology, and movement disorders specialists interchangeable? Mov Disord [Internet]. 2005 Dec [cited 2018 Nov 15];20(12):1577–84. Available from: http://doi.wiley.com/10.1002/mds.20640

7. Mcghee DJM, Ritchie CW, Zajicek JP, Counsell CE. A review of clinical trial designs used to detect a disease-modifying effect of drug therapy in Alzheimer's disease and Parkinson's disease. 2016 [cited 2018 Nov 15];16:92. Available from: http://www.clinicaltrials.gov/

8. Robles-García V, Corral-Bergantiños Y, Espinosa N, Jácome MA, García-Sancho C, Cudeiro J, et al. Spatiotemporal Gait Patterns During Overt and Covert Evaluation in Patients With Parkinson´s Disease and Healthy Subjects: Is There a Hawthorne Effect? J Appl Biomech [Internet]. 2015 Jun [cited 2018 Nov 15];31(3):189–94. Available from: http://journals.humankinetics.com/doi/10.1123/jab.2013-0319

9. Cubo E, Mariscal N, Solano B, Becerra V, Armesto D, Calvo S, et al. Prospective study on cost-effectiveness of home-based motor assessment in Parkinson's disease. J Telemed Telecare [Internet]. 2017 Feb 8 [cited 2019 Nov 26];23(2):328–38. Available from: http://journals.sagepub.com/doi/10.1177/1357633X16638971

10. Duncan RP, Earhart GM. Measuring participation in individuals with Parkinson disease: Relationships with disease severity, quality of life, and mobility. Disabil Rehabil. 2011;33(15–16):1440–6.

11. Silva de Lima AL, Smits T, Darweesh SKL, Valenti G, Milosevic M, Pijl M, et al. Home-based monitoring of falls using wearable sensors in Parkinson's disease. Mov Disord. 2019;35:109–15.

12. Van Uem JMT, Isaacs T, Lewin A, Bresolin E, Salkovic D, Espay AJ, et al. A viewpoint on wearable technology-enabled measurement of wellbeing and health-related quality of life in Parkinson's disease. Vol. 6, Journal of Parkinson's Disease. IOS Press; 2016. p. 279–87.

13. Godinho C, Domingos J, Cunha G, Santos AT, Fernandes RM, Abreu D, et al. A systematic review of the characteristics and validity of monitoring technologies to assess Parkinson's



For peer review only

For peer review only - http://bmjopen.bmj.com/site/about/guidelines.xhtml

UOB Confidential

disease. J Neuroeng Rehabil. 2016;13:24.

14. Sánchez-Ferro Á, Elshehabi M, Godinho C, Salkovic D, Hobert MA, Domingos J, et al. New methods for the assessment of Parkinson's disease (2005 to 2015): A systematic review. Mov Disord. 2016;31:1283–92.

15. Griffiths RI, Kotschet K, Arfon S, Xu ZM, Johnson W, Drago J, et al. Automated assessment of bradykinesia and dyskinesia in Parkinson's disease. J Parkinsons Dis [Internet]. 2012 [cited 2019 Aug 15];2(1):47–55. Available from: http://www.ncbi.nlm.nih.gov/pubmed/23939408

16. Dranca L, de Abetxuko Ruiz de Mendarozketa L, Goñi A, Illarramendi A, Navalpotro Gomez I, Delgado Alvarado M, et al. Using Kinect to classify Parkinson's disease stages related to severity of gait impairment. BMC Bioinformatics. 2018 Dec 10;19(1).

17. Artusi CA, Mishra M, Latimer P, Vizcarra JA, Lopiano L, Maetzler W, et al. Integration of technology-based outcome measures in clinical trials of Parkinson and other neurodegenerative diseases. Parkinsonism Relat Disord [Internet]. 2018 Jan [cited 2018 Nov 15];46 Suppl 1:S53–6. Available from: https://linkinghub.elsevier.com/retrieve/pii/S1353802017302699

18. Espay AJ, Hausdorff JM, Sánchez-Ferro Á, Klucken J, Merola A, Bonato P, et al. A roadmap for implementation of patient-centered digital outcome measures in Parkinson's disease obtained using mobile health technologies. Mov Disord [Internet]. 2019 May 22 [cited 2019 Aug 18];34(5):657–63. Available from: http://www.ncbi.nlm.nih.gov/pubmed/30901495

19. Ellis T, Cavanaugh JT, Earhart GM, Ford MP, Foreman KB, Dibble LE. Which Measures of Physical Function and Motor Impairment Best Predict Quality of Life in Parkinson's Disease? 2011 [cited 2018 Nov 15]; Available from: https://www.ncbi.nlm.nih.gov/pmc/articles/PMC3200468/pdf/nihms316744.pdf

20. Del Din S, Godfrey A, Mazzà C, Lord S, Rochester L. Free-living monitoring of Parkinson's disease: Lessons from the field. Mov Disord. 2016;31(9):1293–313.

21. Woznowski P, Burrows A, Diethe T, Fafoutis X, Hall J, Hannuna S, et al. SPHERE: A Sensor Platform for Healthcare in a Residential Environment. In: Designing, Developing, and Facilitating Smart Cities [Internet]. Cham: Springer International Publishing; 2017 [cited 2020 Feb 20]. p. 315–33. Available from: http://link.springer.com/10.1007/978-3-319-44924-1_14

22. Zhu N, Diethe T, Camplani M, Tao L, Burrows A, Twomey N, et al. Bridging e-Health and the Internet of Things: The SPHERE Project. IEEE Intell Syst [Internet]. 2015 Jul [cited 2018 Nov 15];30(4):39–46. Available from: http://ieeexplore.ieee.org/document/7156004/

23. Burrows A, Gooberman-Hill R, Coyle D. Empirically derived user attributes for the design of home healthcare technologies. Pers Ubiquitous Comput [Internet]. 2015 Dec 6 [cited 2018 Nov 15];19(8):1233–45. Available from: http://link.springer.com/10.1007/s00779-015-0889-1

24. Burrows A, Gooberman-Hill R, Coyle D. Shared Language and the Design of Home Healthcare Technology. In: Proceedings of the 2016 CHI Conference on Human Factors in Computing Systems - CHI '16 [Internet]. New York, New York, USA: ACM Press; 2016 [cited 2018 Nov 15]. p. 3584–94. Available from: http://dl.acm.org/citation.cfm?doid=2858036.2858496

25. Grant S, Blom AW, Whitehouse MR, Craddock I, Judge A, Tonkin EL, et al. Using home sensing technology to assess outcome and recovery after hip and knee replacement in the UK: The HEmiSPHERE study protocol. BMJ Open. 2018 Jul 1;8(7):e021862.

For peer review only



For peer review only - http://bmjopen.bmj.com/site/about/guidelines.xhtml

UOB Confidential

26. Camplani M, Hannuna S, Mirmehdi M, Damen D, Paiement A, Tao L, et al. Real-time RGB-D Tracking with Depth Scaling Kernelised Correlation Filters and Occlusion Handling. In: Procedings of the British Machine Vision Conference 2015 [Internet]. British Machine Vision Association; 2015 [cited 2018 Nov 15]. p. 145.1-145.11. Available from: http://www.bmva.org/bmvc/2015/papers/paper145/index.html

27. Tao L, Burghardt T, Hannuna S, Camplani M, Paiement A, Damen D, et al. A comparative home activity monitoring study using visual and inertial sensors. In: 2015 17th International Conference on E-health Networking, Application & Services (HealthCom) [Internet]. IEEE; 2015 [cited 2018 Nov 15]. p. 644–7. Available from: http://ieeexplore.ieee.org/document/7454583/

28. Hughes AJ, Daniel SE, Kilford L, Lees AJ. Accuracy of clinical diagnosis of idiopathic Parkinson's disease: A clinico-pathological study of 100 cases. J Neurol Neurosurg Psychiatry. 1992;55(3):181–4.

29. Braun V, Clarke V. Using thematic analysis in psychology. Qual Res Psychol. 2006;3(2):77–101.

30. Rosen LD, Whaling K, Carrier LM, Cheever NA, Rokkum J. The Media and Technology Usage and Attitudes Scale: An empirical investigation. Comput Human Behav. 2013;29(6):2501–11.

For peer review only



UOB Confidential

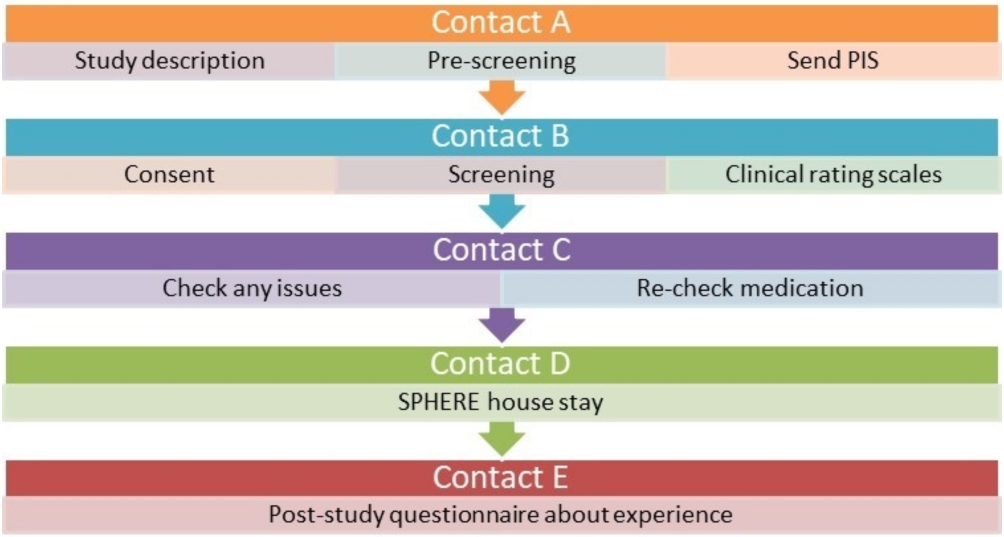


Figure 1. Flow chart detailing outline of PD SENSORS study contacts.
PIS = Participant Information Sheet

159x89mm (120 x 120 DPI)

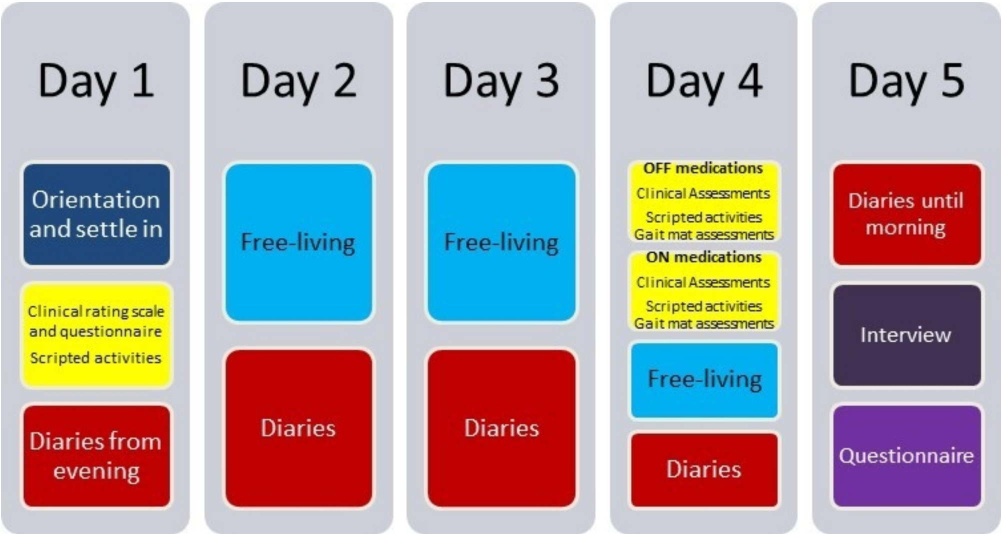


Figure 2. Contact D overview for participants.
OFF medications = the participant with PD in practically-defined 'off' medication state which entails withholding dopaminergic medications for a period of more than 12 hours; ON medications = the participant with PD having taken their dopaminergic medications.

159x84mm (120 x 120 DPI)

**Supplementary Material**

A list of some of the specific movements/actions to be evaluated in the scripted activities during Contact D

- Spreading hummus on bread
- Cutting
- Pouring
- Scooping
- Stirring
- Turning tap on and off
- Wiping surface
- Dispensing soap

For peer review only

# Reporting checklist for protocol of a clinical trial.

Based on the SPIRIT guidelines.

## Instructions to authors

Complete this checklist by entering the page numbers from your manuscript where readers will find each of the items listed below.

Your article may not currently address all the items on the checklist. Please modify your text to include the missing information. If you are certain that an item does not apply, please write "n/a" and provide a short explanation.

Upload your completed checklist as an extra file when you submit to a journal.

In your methods section, say that you used the SPIRITreporting guidelines, and cite them as:

Chan A-W, Tetzlaff JM, Altman DG, Laupacis A, Gøtzsche PC, Krleža-Jerić K, Hróbjartsson A, Mann H, Dickersin K, Berlin J, Doré C, Parulekar W, Summerskill W, Groves T, Schulz K, Sox H, Rockhold FW, Rennie D, Moher D. SPIRIT 2013 Statement: Defining standard protocol items for clinical trials. Ann Intern Med. 2013;158(3):200-207

| | | Reporting Item | Page Number |
|---|---|---|---|
| **Administrative information** | | | |
| Title | #1 | Descriptive title identifying the study design, population, interventions, and, if applicable, trial acronym | 1 |

For peer review only

| | | | |
|---|---|---|---|
| Trial registration | #2a | Trial identifier and registry name. If not yet registered, name of intended registry | 1 |
| Trial registration: data set | #2b | All items from the World Health Organization Trial Registration Data Set | N/A |
| Protocol version | #3 | Date and version identifier | 4 |
| Funding | #4 | Sources and types of financial, material, and other support | 14 |
| Roles and responsibilities: contributorship | #5a | Names, affiliations, and roles of protocol contributors | 14-15 |
| Roles and responsibilities: sponsor contact information | #5b | Name and contact information for the trial sponsor | 15 |
| Roles and responsibilities: sponsor and funder | #5c | Role of study sponsor and funders, if any, in study design; collection, management, analysis, and interpretation of data; writing of the report; and the decision to submit the report for publication, including whether they will have ultimate authority over any of these activities | 15 |
| Roles and responsibilities: committees | #5d | Composition, roles, and responsibilities of the coordinating centre, steering committee, endpoint adjudication committee, data management team, and other individuals or groups overseeing the trial, if applicable (see Item 21a for data | 13, 15 |

For peer review only

| | | | |
|---|---|---|---|
| | | monitoring committee) | |
| **Introduction** | | | |
| Background and rationale | #6a | Description of research question and justification for undertaking the trial, including summary of relevant studies (published and unpublished) examining benefits and harms for each intervention | 3-5 |
| Background and rationale: choice of comparators | #6b | Explanation for choice of comparators | 11 |
| Objectives | #7 | Specific objectives or hypotheses | 5 |
| Trial design | #8 | Description of trial design including type of trial (eg, parallel group, crossover, factorial, single group), allocation ratio, and framework (eg, superiority, equivalence, non-inferiority, exploratory) | 5 |
| **Methods: Participants, interventions, and outcomes** | | | |
| Study setting | #9 | Description of study settings (eg, community clinic, academic hospital) and list of countries where data will be collected. Reference to where list of study sites can be obtained | 5 |
| Eligibility criteria | #10 | Inclusion and exclusion criteria for participants. If applicable, | 6 |

For peer review only

| | | | |
|---|---|---|---|
| | | eligibility criteria for study centres and individuals who will perform the interventions (eg, surgeons, psychotherapists) | |
| Interventions: description | #11a | Interventions for each group with sufficient detail to allow replication, including how and when they will be administered | 6-9 |
| Interventions: modifications | #11b | Criteria for discontinuing or modifying allocated interventions for a given trial participant (eg, drug dose change in response to harms, participant request, or improving / worsening disease) | N/A |
| Interventions: adherance | #11c | Strategies to improve adherence to intervention protocols, and any procedures for monitoring adherence (eg, drug tablet return; laboratory tests) | N/A |
| Interventions: concomitant care | #11d | Relevant concomitant care and interventions that are permitted or prohibited during the trial | 6 |
| Outcomes | #12 | Primary, secondary, and other outcomes, including the specific measurement variable (eg, systolic blood pressure), analysis metric (eg, change from baseline, final value, time to event), method of aggregation (eg, median, proportion), and time point for each outcome. Explanation of the clinical relevance of chosen efficacy and harm outcomes is strongly recommended | 5, 7, 11-13 |
| Participant timeline | #13 | Time schedule of enrolment, interventions (including any run-ins and washouts), assessments, and visits for participants. A schematic diagram is highly recommended (see Figure) | 7 |

For peer review only

| | | | |
|---|---|---|---|
| Sample size | #14 | Estimated number of participants needed to achieve study objectives and how it was determined, including clinical and statistical assumptions supporting any sample size calculations | 11 |
| Recruitment | #15 | Strategies for achieving adequate participant enrolment to reach target sample size | 6 |
| **Methods: Assignment of interventions (for controlled trials)** | | | |
| Allocation: sequence generation | #16a | Method of generating the allocation sequence (eg, computer-generated random numbers), and list of any factors for stratification. To reduce predictability of a random sequence, details of any planned restriction (eg, blocking) should be provided in a separate document that is unavailable to those who enrol participants or assign interventions | N/A |
| Allocation concealment mechanism | #16b | Mechanism of implementing the allocation sequence (eg, central telephone; sequentially numbered, opaque, sealed envelopes), describing any steps to conceal the sequence until interventions are assigned | N/A |
| Allocation: implementation | #16c | Who will generate the allocation sequence, who will enrol participants, and who will assign participants to interventions | N/A |
| Blinding | #17a | Who will be blinded after assignment to interventions (eg, trial | N/A |

For peer review only

| | | | |
|---|---|---|---|
| (masking) | | participants, care providers, outcome assessors, data analysts), and how | |
| Blinding (masking): emergency unblinding | #17b | If blinded, circumstances under which unblinding is permissible, and procedure for revealing a participant's allocated intervention during the trial | N/A |
| **Methods: Data collection, management, and analysis** | | | |
| Data collection plan | #18a | Plans for assessment and collection of outcome, baseline, and other trial data, including any related processes to promote data quality (eg, duplicate measurements, training of assessors) and a description of study instruments (eg, questionnaires, laboratory tests) along with their reliability and validity, if known. Reference to where data collection forms can be found, if not in the protocol | 7-10 |
| Data collection plan: retention | #18b | Plans to promote participant retention and complete follow-up, including list of any outcome data to be collected for participants who discontinue or deviate from intervention protocols | 13 |
| Data management | #19 | Plans for data entry, coding, security, and storage, including any related processes to promote data quality (eg, double data | 13 |

For peer review only

| | | | |
|---|---|---|---|
| | | entry; range checks for data values). Reference to where details of data management procedures can be found, if not in the protocol | |
| Statistics: outcomes | #20a | Statistical methods for analysing primary and secondary outcomes. Reference to where other details of the statistical analysis plan can be found, if not in the protocol | 11-13 |
| Statistics: additional analyses | #20b | Methods for any additional analyses (eg, subgroup and adjusted analyses) | 11-13 |
| Statistics: analysis population and missing data | #20c | Definition of analysis population relating to protocol non-adherence (eg, as randomised analysis), and any statistical methods to handle missing data (eg, multiple imputation) | N/A |
| **Methods: Monitoring** | | | |
| Data monitoring: formal committee | #21a | Composition of data monitoring committee (DMC); summary of its role and reporting structure; statement of whether it is independent from the sponsor and competing interests; and reference to where further details about its charter can be found, if not in the protocol. Alternatively, an explanation of why a DMC is not needed | 12 |
| Data monitoring: interim analysis | #21b | Description of any interim analyses and stopping guidelines, including who will have access to these interim results and | N/A |

For peer review only

| | | | |
|---|---|---|---|
| | | make the final decision to terminate the trial | |
| Harms | #22 | Plans for collecting, assessing, reporting, and managing solicited and spontaneously reported adverse events and other unintended effects of trial interventions or trial conduct | 9-10 |
| Auditing | #23 | Frequency and procedures for auditing trial conduct, if any, and whether the process will be independent from investigators and the sponsor | 13 |
| **Ethics and dissemination** | | | |
| Research ethics approval | #24 | Plans for seeking research ethics committee / institutional review board (REC / IRB) approval | 6 |
| Protocol amendments | #25 | Plans for communicating important protocol modifications (eg, changes to eligibility criteria, outcomes, analyses) to relevant parties (eg, investigators, REC / IRBs, trial participants, trial registries, journals, regulators) | 11 |
| Consent or assent | #26a | Who will obtain informed consent or assent from potential trial participants or authorised surrogates, and how (see Item 32) | 8 |
| Consent or assent: ancillary studies | #26b | Additional consent provisions for collection and use of participant data and biological specimens in ancillary studies, if applicable | N/A |
| Confidentiality | #27 | How personal information about potential and enrolled participants will be collected, shared, and maintained in order | 14 |

For peer review only

| | | | |
|---|---|---|---|
| | | to protect confidentiality before, during, and after the trial | |
| Declaration of interests | #28 | Financial and other competing interests for principal investigators for the overall trial and each study site | 14 |
| Data access | #29 | Statement of who will have access to the final trial dataset, and disclosure of contractual agreements that limit such access for investigators | 13 |
| Ancillary and post trial care | #30 | Provisions, if any, for ancillary and post-trial care, and for compensation to those who suffer harm from trial participation | N/A |
| Dissemination policy: trial results | #31a | Plans for investigators and sponsor to communicate trial results to participants, healthcare professionals, the public, and other relevant groups (eg, via publication, reporting in results databases, or other data sharing arrangements), including any publication restrictions | 13-14 |
| Dissemination policy: authorship | #31b | Authorship eligibility guidelines and any intended use of professional writers | N/A |
| Dissemination policy: reproducible research | #31c | Plans, if any, for granting public access to the full protocol, participant-level dataset, and statistical code | 13 |
| **Appendices** | | | |
| Informed consent materials | #32 | Model consent form and other related documentation given to participants and authorised surrogates | Appendix |

For peer review only

| | | | |
|---|---|---|---|
| Biological specimens | #33 | Plans for collection, laboratory evaluation, and storage of biological specimens for genetic or molecular analysis in the current trial and for future use in ancillary studies, if applicable | N/A |

None The SPIRIT checklist is distributed under the terms of the Creative Commons Attribution License CC-BY-ND 3.0. This checklist can be completed online using https://www.goodreports.org/, a tool made by the EQUATOR Network in collaboration with Penelope.ai

For peer review only

BMJ Open

# Protocol for PD SENSORS: Parkinson's Disease Symptom Evaluation in a Naturalistic Setting producing Outcomes measuRes using SPHERE technology. An observational feasibility study of multi-modal multi-sensor technology to measure symptoms and activities of daily living in Parkinson's disease.



| | |
|---|---|
| Journal: | *BMJ Open* |
| Manuscript ID | bmjopen-2020-041303.R1 |
| Article Type: | Protocol |
| Date Submitted by the Author: | 01-Oct-2020 |





| | |
|---|---|
| Complete List of Authors: | Morgan, Catherine; University of Bristol Medical School, Translational Health Sciences; North Bristol NHS Trust, Movement Disorders Group,<br>Craddock, Ian ; University of Bristol, School of Computer Science, Electrical and Electronic Engineering, and Engineering Mathematics, Faculty of Engineering<br>Tonkin, Emma; University of Bristol, School of Computer Science, Electrical and Electronic Engineering, and Engineering Mathematics, Faculty of Engineering<br>Kinnunen, Kirsi; IXICO plc<br>McNaney, Roisin; University of Bristol, School of Computer Science, Electrical and Electronic Engineering, and Engineering Mathematics, Faculty of Engineering<br>Whitehouse, Sam; University of Bristol, School of Computer Science, Electrical and Electronic Engineering, and Engineering Mathematics, Faculty of Engineering<br>Mirmehdi, Majid; University of Bristol, School of Computer Science, Electrical and Electronic Engineering, and Engineering Mathematics, Faculty of Engineering<br>Heidarivincheh, Farnoosh; University of Bristol, School of Computer Science, Electrical and Electronic Engineering, and Engineering Mathematics, Faculty of Engineering<br>McConville, Ryan; University of Bristol, School of Computer Science, Electrical and Electronic Engineering, and Engineering Mathematics, Faculty of Engineering<br>Carey, Julia; University of Bristol, School of Computer Science, Electrical and Electronic Engineering, and Engineering Mathematics, Faculty of Engineering<br>Horne, Alison; University of Bristol Medical School<br>Rolinski, Michal; University of Bristol Medical School, Translational Health Sciences; North Bristol NHS Trust, Movement Disorders Group,<br>Rochester, Lynn; Newcastle University, Institute of Neuroscience; Newcastle Upon Tyne Hospitals NHS Foundation Trust<br>Maetzler, Walter; University Medical Center Schleswig-Holstein Campus Kiel, Department of Neurology<br>Matthews, Helen; Cure Parkinson's Trust |

| | |
|---|---|
| | Watson, Oliver; Bristol Health Partners<br>Eardley, Rachel; University of Bristol, School of Computer Science, Electrical and Electronic Engineering, and Engineering Mathematics, Faculty of Engineering<br>Whone, Alan; University of Bristol , School of Translational Health Sciences ; North Bristol NHS Trust, Movement Disorders Group |
| <b>Primary Subject Heading</b>: | Neurology |
| Secondary Subject Heading: | Research methods |
| Keywords: | Parkinson-s disease < NEUROLOGY, Information technology < BIOTECHNOLOGY & BIOINFORMATICS, QUALITATIVE RESEARCH, STATISTICS & RESEARCH METHODS |
| | |

SCHOLARONE™
Manuscripts

*I, the Submitting Author has the right to grant and does grant on behalf of all authors of the Work (as defined in the below author licence), an exclusive licence and/or a non-exclusive licence for contributions from authors who are: i) UK Crown employees; ii) where BMJ has agreed a CC-BY licence shall apply, and/or iii) in accordance with the terms applicable for US Federal Government officers or employees acting as part of their official duties; on a worldwide, perpetual, irrevocable, royalty-free basis to BMJ Publishing Group Ltd ("BMJ") its licensees and where the relevant Journal is co-owned by BMJ to the co-owners of the Journal, to publish the Work in this journal and any other BMJ products and to exploit all rights, as set out in our licence.*

*The Submitting Author accepts and understands that any supply made under these terms is made by BMJ to the Submitting Author unless you are acting as an employee on behalf of your employer or a postgraduate student of an affiliated institution which is paying any applicable article publishing charge ("APC") for Open Access articles. Where the Submitting Author wishes to make the Work available on an Open Access basis (and intends to pay the relevant APC), the terms of reuse of such Open Access shall be governed by a Creative Commons licence – details of these licences and which Creative Commons licence will apply to this Work are set out in our licence referred to above.*

*Other than as permitted in any relevant BMJ Author's Self Archiving Policies, I confirm this Work has not been accepted for publication elsewhere, is not being considered for publication elsewhere and does not duplicate material already published. I confirm all authors consent to publication of this Work and authorise the granting of this licence.*

For peer review only

**Protocol for PD SENSORS: Parkinson's Disease Symptom Evaluation in a Naturalistic Setting producing Outcomes measuRes using SPHERE technology. An observational feasibility study of multi-modal multi-sensor technology to measure symptoms and activities of daily living in Parkinson's disease.**

Authors: Catherine Morgan [a,c,*] , Ian Craddock [b], Emma Tonkin [b], Kirsi M Kinnunen [d], Roisin McNaney [b], Sam Whitehouse [b], Majid Mirmehdi [b], Farnoosh Heidarivincheh [b], Ryan McConville [b], Julia Carey [b], Alison Horne [e], Michal Rolinski [a,c], Lynn Rochester [f,g], Walter Maetzler [h], Helen Matthews [i], Oliver Watson [j], Rachel Eardley [b], Alan L Whone [a,c]

[a] University of Bristol Medical School, Translational Health Sciences, Bristol, UK

[b] University of Bristol, School of Computer Science, Electrical and Electronic Engineering, and Engineering Mathematics, Faculty of Engineering, Bristol, UK

[c] North Bristol NHS Trust, Movement Disorders Group, Westbury on Trym, Avon, UK

[d] IXICO plc, London, UK

[e] University of Bristol Medical School, Bristol, UK

[f] Newcastle University, Institute of Neuroscience, Newcastle, UK

[g] Newcastle Upon Tyne Hospitals NHS Foundation Trust, Newcastle Upon Tyne, UK

[h] University Medical Center Schleswig-Holstein Campus Kiel, Department of Neurology, Kiel, Schleswig-Holstein, DE

[i] Cure Parkinson's Trust, London, UK

[j] Bristol Health Partners, Bristol, UK

**Emails of authors**: Catherine.morgan@bristol.ac.uk; ian.craddock@bristol.ac.uk; e.l.tonkin@bristol.ac.uk; kirsi.kinnunen@ixico.com; r.mcnaney@bristol.ac.uk; sw12690@bristol.ac.uk; m.mirmehdi@bristol.ac.uk; farnoosh.heidarivincheh@bristol.ac.uk; ryan.mcconville@bristol.ac.uk; Julia.carey@bristol.ac.uk; alison.horne@bristol.ac.uk; michal.rolinski@bristol.ac.uk; lynn.rochester@newcastle.ac.uk; w.maetzler@neurologie.uni-kiel.de; helen@cureparkinsons.org.uk; oliver.watson@bristol.ac.uk; Rachel.eardley@bristol.ac.uk; alan.whone@bristol.ac.uk

**Correspondence to:** Dr Catherine Morgan; Catherine.morgan@bristol.ac.uk; Movement Disorders Group, Bristol Brain Centre, Southmead Hospital, North Bristol National Health Service Trust, Bristol, UK; +44 7970 657958

**Registration:** University of Bristol Research Registration Database, reference 2018-4247

**Keywords:** Parkinson's disease; Information technology; Qualitative research; Statistics and research methods

For peer review only



UOB Confidential

**Word count:** 5837

**Abstract**

Introduction: The impact of disease-modifying agents on disease progression in Parkinson's disease is largely assessed in clinical trials using clinical rating scales. These scales have drawbacks in terms of their ability to capture the fluctuating nature of symptoms while living in a naturalistic environment. The SPHERE (Sensor Platform for HEalthcare in a Residential Environment) project has designed a multi-sensor platform with multimodal devices designed to allow continuous, relatively inexpensive, unobtrusive sensing of motor, non-motor and activities of daily living metrics in a home or a home-like environment. The aim of this study is to evaluate how the SPHERE technology can measure aspects of Parkinson's disease.

Methods and Analysis: This is a small-scale feasibility and acceptability study during which 12 pairs of participants (comprising a person with Parkinson's and a healthy control participant) will stay and live freely for 5 days in a home-like environment embedded with SPHERE technology including environmental, appliance monitoring, wrist-worn accelerometry and silhouette video sensors. These data will be collected alongside clinical rating scales, participant diary entries and expert clinician annotations of colour video images. Machine learning will be used to look for a signal to discriminate between Parkinson's disease and control, and between Parkinson's disease symptoms 'on' or 'off' medications. Additional outcome measures including bradykinesia, activity level, sleep parameters and some activities of daily living will be explored. Acceptability of the technology will be evaluated qualitatively using semi-structured interviews.

Ethics and Dissemination: Ethical approval has been given to commence this study; the results will be disseminated as widely as appropriate.

Strengths of this study:

- The location for testing (SPHERE house) allows free living whilst providing some ground truth through colour video capture of participants' activities for clinician annotation.
- Due to the multi-sensor data collection platform within the SPHERE house, the performance of machine learning models which are based on single or multiple modalities could be assessed.
- The qualitative tools will add the important dimension of the person with Parkinson's perspective on this technology.

Limitations of this study:

- The small number of participants means that results generated from this study will not be generalizable to the wider population of people with PD.
- The feasibility nature of PD SENSORS means that cost effectiveness will not be explored at this stage.

**Introduction**

Parkinson's disease (PD) is the second most common neurodegenerative disease in the UK, affecting more than 127,000 people. It is a chronic, progressive, disabling disease which is characterised by a



For peer review only - http://bmjopen.bmj.com/site/about/guidelines.xhtml

UOB Confidential

For peer review only

large variety of possible motor symptoms (including bradykinesia, rigidity and tremor) and non-motor symptoms (including sleep disturbance, cognitive impairment and pain) (1). It is estimated that by the time of diagnosis with PD most patients have already lost around 50% of their dopaminergic neurones (2), therefore attempts to modify the disease should be aimed at pre-clinical or early-stage disease states.

The search for a treatment which could modify PD, a so-called disease-modifying therapy or 'DMT', has thus far proven fruitless with no DMT reaching market authorisation. This is despite multiple potential therapies having been tested in randomised, double-blind clinical trials (3).

Clinical trials testing DMTs have often used a clinical rating scale to quantify symptoms and monitor response to treatment as a primary outcome measure (3). The gold-standard rating scale is the Movement Disorder Society-sponsored revision of the Unified Parkinson's Disease Rating Scale (MDS-UPDRS) (4). This is a validated (5) tool which evaluates a comprehensive spectrum of symptoms and activities. However it is also a subjective (6) and non-linear (7) tool which exposes the study participant to the risk of observer bias and the 'Hawthorne Effect' (8). It is used episodically, giving a snapshot of a person with PD, which may not capture the day-to-day and hour-to-hour fluctuations of this complex disease. The testing location is often an artificial environment like a clinic or laboratory. This can increase costs (9) and impair the ability of studies to appraise important activities of daily living (ADLs) such as engagement in social activities (10), rare events such as falls (11) and other metrics which affect wellbeing and quality of life such as sleep (12).

For peer review only

Development of technologies for the measurement of various aspects of PD have been evolving over the past 15 years (13). Particularly motor symptoms, including gait and bradykinesia, are amenable to measurement by technology (14) continuously and over long time periods. For example, the Parkinson's KinetiGraph wearable movement recording device (developed by Global Kinetics) can measure tremor, bradykinesia and dyskinesia(15) and the Microsoft Kinect motion sensor can classify PD stages relating to gait impairment (16). However, these tools are still only partially integrated into clinical trials, with most continuing to use periodic clinical rating scale assessments in the non-ecological setting as primary or secondary end-points (17). There are excellent initiatives which are guiding this field forward, for example a 'roadmap' for implementation of digital outcome measures using technologies (18), which are tackling the important questions surrounding regulatory approvals and collaboration with industry.

Lacunae in the literature about technology-assisted outcomes in PD include the measurement of non-motor outcomes (14) such as constipation and activities of daily living. The frequent use of wearable devices which are attached to specific body parts means that the information gathered is restricted to the movement in that part of the body; this may be inadequate fully to understand the context in which a measurement is taken and may result in low correlations with measures of functional disability and/or quality of life (19). In addition, it can be challenging to establish and operationalise the 'ground truth' for benchmarking novel measurements. Reviews of technology-assisted outcome measures in a free-living environment have highlighted that to date there is no fully-validated system which evaluates clinical features of PD in a naturalistic/ecologically valid (i.e. home or home-like) environment (20).

**Sensor Platform for HEalthcare in a Residential Environment - SPHERE**



For peer review only - http://bmjopen.bmj.com/site/about/guidelines.xhtml

UOB Confidential

The long-standing SPHERE project from the University of Bristol (SPHERE: Sensor Platform for HEalthcare in a Residential Environment) has led to the design of a multi-sensor platform with multimodal devices designed to allow continuous, relatively inexpensive, unobtrusive sensing of motor, non-motor and ADL metrics in a home or a home-like environment (21,22).

The SPHERE sensor platform is embedded into a test-bed location called the 'SPHERE house'. This is a 2-bedroom, 2-storey property with kitchen, living room, dining room, bathroom, and toilet.

Within the SPHERE house there are a number of sensors which include: wearable devices with accelerometers (in-house developed); environmental/ambient sensors which measure temperature, humidity and light levels; RGB-D ('red green blue – depth', referring to the three colour planes that together can generate the colour of a pixel in an image, in addition to the recording of distances to surfaces using infra-red light) camera sensors that generate silhouettes of participants and bounding boxes that, when linked with the SPHERE wearable, let the system know who the participant is; appliance sensors which are able to tell when and how frequently an appliance is switched on and off; PIR (Passive InfraRed) sensors which detect motion in each room.

The SPHERE sensor platform has undergone extensive testing with healthy volunteers, being deployed to 50 homes over a year from January 2017. High-quality qualitative work has been conducted looking at the design and acceptability of the SPHERE sensors and technology in a person's life (23,24). In addition, the SPHERE platform is being utilised in 3 clinical projects which are at different stages of completion, looking at outcomes around cardiac valve replacement, hip and knee surgery (25) and in dementia/mild cognitive impairment.

The multimodal multi-sensor design of the SPHERE system (22) enables a richer and more fine-grained picture of free-living symptoms and activities to be collected. For example, the combined use of the wearable devices with accelerometers, alongside the video and environmental sensors, enables machine learning algorithms to opportunistically and continuously fuse data to build a time series of activities of daily living for each person in the home (26,27). This offers an exciting opportunity to conduct free-living evaluation of people with PD.

**Parkinson's Disease Symptom Evaluation in a Naturalistic Setting producing Outcomes measuRes using SPHERE technology – PD SENSORS**

Given the above gaps in knowledge, and appreciating the SPHERE system's capability, it is believed that SPHERE technology could be exploited for filling some of the gaps in the current use of technology to measure outcomes in PD (e.g. for non-motor symptoms and ADLs). SPHERE technology has the potential to complement and enhance the existing clinical rating scales used to evaluate Parkinson's disease. PD SENSORS is a feasibility and acceptability study using the SPHERE platform to develop novel outcome measures in PD. It is designed with the intent that it will inform a future larger scale and longer-term investigation, in which SPHERE technology will be deployed to multiple homes of people with PD (protocol version 1.3; August 2020). This is a truly collaborative study with not only strong interdisciplinary collaboration between computer scientists and movement disorders clinicians but also industry involvement. The university academic team are working with a UK-based imaging and digital biomarker analysis company, IXICO, that contributes clinical and data science expertise and is providing additional wearable devices from Axivity (AX3)



For peer review only - http://bmjopen.bmj.com/site/about/guidelines.xhtml

UOB Confidential

For peer review only

and Activinsights (GENEActiv Original) and gait mat technology from ProtoKinetics (Zeno instrumented walkway), to further enrich the dataset.

**Aims**

**Primary aims**

To understand how the raw sensor data obtained from the SPHERE system can be processed to translate into meaningful data for the clinical scientist on metrics of:

- Total body movement
- Bradykinesia
- Sleep quantity and quality
- Urinary dysfunction
- Meal preparation/consumption
- Cleaning tasks
- Getting out of a chair
- Activity level (ambulatory activity and sedentary behaviour)
- Room to room transfers and time spent in each room

**Secondary aims**

1. To evaluate the acceptability of the continuous use of multiple and varied SPHERE sensors over a 5-day period in a home-like setting for people with Parkinson's disease and to explore the participants' experience of such a study
2. To explore the use of home sensing as a means to discriminate between participants with PD and healthy control participants
3. To assess the criterion validity of the technology-assisted outcomes by mapping the sensor data outcomes to the gold-standard clinical rating scale (MDS-UPDRS) and patient-reported outcome measure (PDQ-39) when participants with PD are in both 'on' and 'off' medication states

**Methods**

**Design**

A small-scale feasibility and acceptability study.

**Setting and participants**

The setting will be the SPHERE house: a customised 2-bedroom residential house in Bristol, UK, with multiple embedded sensors and data collection tools.

12 pairs of participants comprising a person with PD and a control participant (likely to be a spouse, other family member, friend, or carer) will be recruited to stay in the SPHERE house for 5 consecutive days.

Participants with PD will be eligible to enter the study if they meet certain inclusion criteria, including:



For peer review only - http://bmjopen.bmj.com/site/about/guidelines.xhtml

UOB Confidential

For peer review only

- Diagnosis of idiopathic Parkinson's disease according to UK Brain Bank Criteria (28);
- Age over 18
- Modified Hoehn and Yahr Scale score of 3 or less in 'off' state (i.e. when they withhold dopaminergic medications)

Inclusion criteria for control participants includes:

- No history of Parkinson's disease, REM (rapid eye movement) sleep behaviour disorder, dementia, or other neurodegenerative/significant musculoskeletal condition

The study's exclusion criteria include:

- Montreal Cognitive Assessment Score < 26
- Current active depressive symptoms; Beck Depression Inventory II score >19
- Use of walking aids whilst inside a house to aid mobility either 'on' or 'off' medications

Exclusion criteria were largely chosen to reduce the potential impact on sensor data of other factors, such as mobility aids altering accelerometry data making it difficult to evaluate bradykinesia.

Participants will be recruited through movement disorders specialist or general neurology outpatient clinics in North Bristol NHS Trust and through posters in the outpatient department of North Bristol NHS Trust, The Cure Parkinson's Trust, a local Patient and Public Involvement Group or by word-of-mouth.

The medical PD care for those study participants with the disease will continue unchanged.

The study will be conducted in accordance with the Declaration of Helsinki and Good Clinical Practice Guidelines. Written informed consent will be obtained from all study participants before any study-related procedures are performed.

Full approval from the National Health Service (NHS) Wales Research Ethics Committee 6 was granted on 17th December 2019, and Health Research Authority and Health and Care Research Wales approval confirmed on 14th January 2020.

Due to delays relating to the COVID-19 pandemic, active study data gathering is planned to commence in October 2020 and, if uninterrupted further, should be complete within 12 months.

**Overview of study contacts**

PD SENSORS is split into 5 study 'contacts' (stages at which information is transmitted to or gathered from the study participants). A flow chart in Figure 1, below, illustrates the main aims of each study contact.

At each study contact, specific information is gathered from the participants by the research team. Table 1 details the tools to be used during the longer study contacts, Contacts B and D, with the participants. The items in italics in this table apply only to the participant with PD and not to the control participant.

Table 1. Baseline and follow-up data from Contacts B and D



For peer review only - http://bmjopen.bmj.com/site/about/guidelines.xhtml

UOB Confidential

For peer review only

| Time Point | Outcomes Measured |
|---|---|
| **Contact B** | Further demographic information including date of birth and *date of diagnosis of PD*<br>Full screening questions according to inclusion and exclusion criteria<br>*Medication regime and calculation of Levodopa equivalent daily dose*<br>*H&Y score*<br>MOCA<br>BDI II, AS, ESS<br>Timed Up and Go Test<br>*NMSS*<br>*PDSS*<br>*RBD-SQ* |
| **Contact D (the SPHERE house stay)** | *MDS-UPDRS (the motor sub-scale will be performed on multiple occasions, once while 'off' medications)*<br>*PDQ-39*<br>Symptom and activity diary<br>Sleep diary<br>Bladder diary<br>*Medication-taking record*<br>Sensor data from scripted activities<br>Sensor data from free-living<br>Sensor data from wearable devices provided through IXICO<br>Annotations of colour video dataset<br>Gait mat assessments<br>Interview with questions relating to experience of living with SPHERE technology |

*AS = Apathy Scale; BDI II = Beck's Depression Inventory II; ESS = Epworth Sleepiness Scale; H&Y = Hoehn and Yahr; MDS-UPDRS = Movement Disorder Society sponsored revision of the Unified Parkinson's Disease Rating Scale; MOCA = Montreal Cognitive Assessment; NMSS = Non-Motor Symptoms Scale for Parkinson's disease; PDQ-39 = Parkinson's Disease Questionnaire-39; PDSS-2 = Parkinson's Disease Sleep Scale-2; RBD-SQ = REM sleep Behaviour Disorder Screening Questionnaire*

Several clinical rating scales will be completed as described at Contact B. This is with the intent to gain a thorough understanding of the nature of the symptoms experienced by the participants with PD, and to be able to therefore make better sense of the sensor data produced from the SPHERE house stay. The Parkinson's Disease Sleep Scale-2 is a 15-point visual analogue scale that has been validated in PD (29) which allows patients with PD to self-rate and quantify the profile of nocturnal disturbances and sleep disruption being experienced. The Non-Motor Symptoms Scale (NMSS) is a 30-question scale which assesses severity and frequency with which a patient with PD experiences a variety of non-movement related symptoms, such as urinary dysfunction, mood/cognitive symptoms and gastrointestinal tract-associated symptoms (30). The Timed Up and Go (TUG) test (31) is a simple, quick and widely-used clinical performance tool which assesses lower extremity function, mobility and falls risk. The REM sleep Behaviour Disorder Screening Questionnaire (RBD-SQ) is a useful screening tool due to its relatively high sensitivity for REM sleep behaviour disorder (32), which affects a significant proportion of patients with PD and may change the interpretation of the sensor data overnight.

During Contact D, the research team will conduct the Movement Disorder Society sponsored revision of the Unified Parkinson's Disease Rating Scale (MDS-UPDRS) (5) with the participant with Parkinson's. This clinical rating scale has subscales evaluating motor experiences of daily living (part



For peer review only

For peer review only - http://bmjopen.bmj.com/site/about/guidelines.xhtml

UOB Confidential

I), non-motor experiences of daily living (part II), motor examination (part III) and motor complications (part IV). The PDQ-39, also to be used in Contact D, is a self-completed patient-reported outcome measure designed to address aspects of functioning and well-being for those affected by Parkinson's disease (33).

**Schedule of Contact D: the stay in the SPHERE house**

The pair of participants will stay in the house together for five days. The duration of stay was chosen in consultation with people with PD and their acquaintances who took part in pre-study focus groups. During the time they are in the house, the participants will be encouraged to continue living freely and undertake their regular activities of daily living as normally as possible.

Figure 2 shows a day-by-day plan for Contact D for the pair of participants. The figure illustrates that the stay in the SPHERE house for each pair of participants will also involve:

- Researchers will visit the house on two occasions, Day 1 and Day 4, to conduct scripted activities and clinical rating scales with participants.
- During Days 2 and 3 the participants will live freely with no researcher visits.

Important elements during the 5-day stay are:

- The participant with PD will withhold their dopaminergic medications for >12 hours so that they are in the practically-defined 'off' medication state. During this time 'off' medications (on Day 4), some assessments will be undertaken including the MDS-UPDRS III (motor subsection). These assessments will then be repeated after the participant with PD restarts their medications that day.
- Specific food preparation and cleaning/hygiene scripted activities, will be witnessed and annotated by a study team member during their planned visits to the house. The participant with PD will undertake these activities on Day 1 and twice on Day 4, first 'off' then 'on' medications, whilst the control participant will do the activities once each on Day 1 and Day 4. More detail about these activities is shown in the supplementary material.
- Wearable devices: in addition to the SPHERE wearable device on each wrist, an extra IXICO-provided wearable will be worn on each wrist throughout the SPHERE house stay. During the clinical assessments and scripted activities on day 4 one additional wearable accelerometer will be placed around the trunk to locate in the lower back (lumbar) area and another one on a lower limb (shin).

Gait mat assessments

During the 'off' and 'on' medication testing session on Day 4, the participants will undertake clinical assessments including an evaluation of gait on the Zeno Walkway, a gait mat embedded with sensors that is placed in the entrance area of SPHERE house where these assessments are conducted. The item is administered according to the standard instructions and the gait mat protocol. All gait mat protocols for these assessments have been developed by ProtoKinetics, the Zeno walkway manufacturers.

Colour video capture



For peer review only



UOB Confidential

In communal areas of the SPHERE house (i.e. not in bedrooms or bathrooms) cameras will be enabled to produce RGB data for the purposes of allowing more accurate and relevant evaluation of the participants' movements. Short periods of these data, collected both while participants are free-living and during clinical assessments, will be used to provide a labelled dataset for the analytic work-up of total body movement using annotations of these videos relating to the participants' activities, location, and aspects of their Parkinson's disease symptomatology.

All work during the events and circumstances relating to severe acute respiratory syndrome coronavirus (SARS-COV-2)/Coronavirus-19 (COVID-19) will be subject to risk assessment and modification to ensure the study is safe for participants and researchers.

**Statistical analysis**

Study researchers and the study statistician will work collaboratively with the clinicians and SPHERE machine learning team to determine how the SPHERE data can be transformed into usable form. The research team will then aim to ascertain if this data can give us a signal to discriminate between participants with PD and control participants and whether it can give us a signal to discriminate between when a participant with PD is 'on' or 'off' medications. We will compare it to the data produced by the gold-standard clinical rating scales and patient-reported outcome measures. Machine learning (ML) will play a key role in analysing the data.

Existing works have proposed methods based on ML for PD diagnosis and monitoring (please refer to (34) for a detailed review). Using various types of data, such as videos, Inertial Measurement Unit (IMU) and smart-phone or wearable accelerometer data, they model the presence and/or severity of PD via ML algorithms such as Artificial Neural Networks (ANN), Hidden Markov Models (HMM), Support Vector Machines (SVM) and Random Forests (RF). We also aim to use the data collected from the SPHERE house, which is a multi-sensor platform, with various ML techniques to assess the reliability of an automatic learning approach for modelling and analysing PD. As mentioned before, the data that will be utilised to learn from includes the output of wearable accelerometer, RGB-D cameras with silhouette extraction, PIR and environmental sensors.

To achieve such analysis, multiple potential approaches could be considered for future work, some of which are presented in the following:

**PD vs non-PD recognition –** Given the input data corresponding to different time scales, such as seconds, minutes, or hours, ML-based model could be trained to discriminate between PD and non-PD (control) subjects. Such recognition could be performed as binary classification or as a probability estimation per category. Further, the predictions corresponding to different durations could be compared to determine the one by which the most reliable recognition is achieved.

**PD severity estimation –** Based on different rating scales such as the MDS-UPDRS or H&Y score, an ML-based approach could learn to estimate the severity of the disease among the PD patients. The ground-truth scores are provided by the clinicians during both 'on' and 'off' medication periods. Then, a model could be trained to regress predictions using the ground-truth scores.

**Fine-grained analysis of PD symptoms –** In addition to PD recognition and severity estimation, an ML-based model could be trained to detect different PD symptoms, such



For peer review only

For peer review only - http://bmjopen.bmj.com/site/about/guidelines.xhtml

UOB Confidential

as bradykinesia or tremor. Also, a finer-grained analysis of each symptom could be investigated. For example, a model could learn to detect peaks or changes during tremor.

**Activity recognition –** The learning could be extended towards recognising the category of the activities which are performed by the subjects during free living or scripted activity experiments. Then, the quality of the performed activities such as meal preparation or cleaning, could be assessed. Also, the relationship between PD symptoms like bradykinesia, and the performed activities could be modelled

**Behaviour analysis of the PD patients –** ML algorithms could be applied to model the behavioural patterns of PD vs non-PD subjects. Such models could be also trained to distinguish between the behaviour of the PD patients during 'on' and 'off' medication periods. Moreover, the patterns in the sleeping habits, mobility, and urinary function of the patients could be learned and analysed.

**Modelling the progression of PD temporally –** The above-mentioned predictions could be monitored through time by modelling the progression of the disease via ML algorithms that provide temporal analysis of the input data. Specifically, models like HMMs or Recurrent Neural Networks (RNNs), which can produce predictions per time point, could be trained to temporally model the PD symptoms. Therefore, for example, PD symptoms could be assessed at different times of the day during the study.

**Analysing the information collected from screening and diaries –** The questionnaires and tests performed during screening (Contact B), as well as the diaries filled by the patients during their stay in the SPHERE house, would provide information which could be summarised via descriptive statistics. Additionally, associations with the data collected from various sensors, for example, environmental sensors, could be modelled via ML.

**Evaluating traditional vs state-of-the-art ML algorithms –** As presented in (34), there are many ML algorithms which can be applied to model PD. We would have the ability to assess the performance of such alternative algorithms. Specifically, a comparison between classical ML algorithms, such as SVMs or RFs, against state-of the-art deep learning-based approaches for modelling PD is of interest, as the latter has achieved high performance in areas such as computer vision.

**Evaluating single vs multi-modal approaches –** Due to the multi-sensor data collection platform within the SPHERE house, the performance of ML models which are based on single or multiple modalities could be assessed. In a multi-modal approach, methods for fusing data from different modalities, e.g. early or late fusion methods, could be also evaluated to determine which modalities are most effective.

**Collaboration with IXICO plc**

The University of Bristol and IXICO plc will collaborate on specific outcomes, as well as through the sharing of data and results, the loan of the wearable biosensors (Axivity AX3 and GENEActiv Original) and gait mat (Zeno walkway) and in-kind contributions of scientific expertise and study operations. The topics explored within this collaboration will include the feasibility of labelling total body bradykinesia from colour video, to developed automated detection of bradykinesia from biosensor data, biosensor device comparability, and relationships between sensor-based measurements from



For peer review only

For peer review only - http://bmjopen.bmj.com/site/about/guidelines.xhtml

UOB Confidential

the gait mat and wearable devices and between sensor-based measurements and traditional clinical measures of motor symptoms, in addition to ADL detection from wearable sensor data.

**Qualitative work**

A semi-structured interview will be conducted with each participant at the end of the study exploring their experience of the SPHERE technology, with a focus on the tolerability and acceptability of such technology for people with PD and those close to them. An interview topic guide is shown in Box 1. Interview transcripts will be read, re-read, and the data gathered will be analysed thematically, using an inductive approach, with methods drawn from Braun and Clarke (35).

Box 1. Interview Topic Guide

- Background experience with technology
- Experience of the SPHERE sensor platform
- Thoughts about the sensor data collected
- The study information received relating to the SPHERE technology
- Questions about the study experience specific to PD and those close to people with PD

Additionally, two questionnaires will be used to augment the qualitative data produced from the interviews. The first questionnaire will be a standardised tool evaluating the participants' attitudes towards and experience of technology: using four attitude-based subscales (looking at positive attitudes, negative attitudes, technological anxiety/dependence, and attitudes towards task-switching) from The Media and Technology Usage and Attitudes Scale (36). This will allow us to interpret the interview responses in the context of a participant's prior background exposure to technology. The second questionnaire comprises three short questions exploring feedback about the sensors or study in general regarding thoughts that could have occurred to the participant in the days after they left the SPHERE house. These questions are listed in Box 2. This enables us to ensure we understand the experience of this sensor platform as fully as possible.

Box 2. Questions following-up the SPHERE house stay (Contact E questionnaire)

1. Following the PD SENSORS study, now that you have finished the study and been at home for a while, do you have any further thoughts to feedback to us about the SPHERE sensors that you lived with for 5 days?
2. Specifically, in relation to your PD/supporting role for a person with PD, was there anything further you wanted us to know about or understand?
3. Is there anything else at all that you felt important to mention to us regarding your study experience?

**Safety reporting**

Any adverse event (AE) will be assessed and acted upon by the research team. All AEs will be recorded in the study or project file with a note that will identify when the event occurred, the details of the AE, any potential study relation, action taken and resolution / closure of the AE. An



For peer review only - http://bmjopen.bmj.com/site/about/guidelines.xhtml

UOB Confidential

assessment of seriousness will be made by the researcher and serious adverse events (SAEs) will be reported in line with current legislation and university guidance.

In addition, all SAEs will be reported to the NHS Research Ethics Committee in the Annual Progress Report.

**Amendments**

Any amendments to protocol design will be communicated to relevant parties including North Bristol NHS Trust and the Research Ethics Committee.

**Sample size**

This sample size (24) was chosen as a balance between gathering enough data from the SPHERE sensors to create excellent quality algorithms (in consultation with our Machine Learning team) and the need to pragmatically take into account the nature of this exploratory study which is developing analytics to use in deployment to people's own homes in a future study. No conventional sample size calculation has been undertaken since we have no clear effect estimates to use in any such calculation; we view this more as a pilot and a proof of concept study. The comparator of the healthy volunteer control participant was chosen in order to make the study more sociable and therefore hopefully enjoyable to the person with PD, alongside also providing study data from people without PD so that conclusions could be drawn about how to identify and then measure aspects of PD.

**Data management and security**

The day-to-day management of the study including data management will be coordinated by the Principal Investigator of the study. All the data collected will be fully anonymised or pseudonymised where possible. Unique study identification numbers will be assigned to each participant. Access to data will be limited to SPHERE researchers and collaborators only, who must be registered (to use the data) with managed access and permissions. Directly identifiable data (e.g. name, address, telephone number) will be held securely and separately from other study data. Data procedures will be in accordance with the General Data Protection Regulation. If a participant withdraws, the data that is already obtained will be kept by the study team. Under a Service Level Agreement between the University of Bristol and University Hospitals Bristol NHS Foundation Trust, the Trust will monitor 10% of the University's Sponsored studies. In line with the National Institute for Health and Research guidance which encourages sharing anonymised datasets, we will seek informed consent from participants for their anonymous data to be shared with other researchers. The data management will be reviewed on a regular basis by an in-house Data Monitoring Committee consisting of data experts, engineers and movement disorders clinicians.

**Patient and public involvement**

The Bristol Movement Disorders Health Integration Team (MOVE-HIT) has an active patient and public involvement (PPI) group which includes people with Parkinson's disease and their partners/carers. The MOVE-HIT PPI group meets regularly and has been involved in providing extensive advice around the study design, as well as the development of the participant information sheets, consent forms and participant diaries. The opinions of members of this PPI group on study



For peer review only - http://bmjopen.bmj.com/site/about/guidelines.xhtml

UOB Confidential

For peer review only

design have been collected both in face-to-face group and individual consultations and in a survey to wider group members. We plan further ongoing consultation, both during the middle of the study to discuss the group's views on study progress and at the end of the study to consult on the plain English report on the study findings and to identify dissemination outlets.

The PD SENSORS research team are also collaborating with Lyndsey Isaacs, a Trustee of The Cure Parkinson's Trust (CPT), who facilitated the presentation of a PD SENSORS study design survey to the delegates at a CPT Research Update Meeting. This survey was also made more widely available on the CPT website. The research team will have ongoing liaison with CPT representatives during the study to discuss aspects of study management such as dissemination of research findings. The CPT have agreed to be a forum through which to disseminate the study Poster to allow interested persons to express interest in participating in PD SENSORS.

**Ethics and Dissemination**

The SPHERE house has been safety checked by an experienced occupational therapist practitioner with an interest in PD and relevant adaptations made.

It is anticipated that the scientific results of the study and its various elements will be published both via presentation at national or international conferences and through publication in peer-reviewed scientific journals/publications.

**Data Statement**

SPHERE intends to make any research data produced as widely and freely available as possible in a timely and responsible manner. The information would be suitable for sharing as we are developing novel and potentially useful ways to assess PD. However, some portions of research data will not be suitable for sharing, e.g., directly identifiable information such as names and addresses. We will seek consent from participants for pseudonymised subsets of their data to be shared with other researchers. This is in anticipation that data sharing and access to such datasets may become mandatory in the coming years.

Data published by the University of Bristol (UoB) Repository can have open or controlled data access. Published datasets will appear in a standardised, online 'data catalogue' accompanied by its deposit record. These deposits are also made visible to search engines such as Google, to help maximise their visibility and impact. We will also create records of digital and/or non-digital data produced in Pure (http://www.bristol.ac.uk/red/research-policy/pure/).

**Funding**

This work was performed under the SPHERE Next Steps Project funded by the UK Engineering and Physical Sciences Research Council (EPSRC), Grant EP/R005273/1.

This work is supported by the Elizabeth Blackwell Institute for Health Research, University of Bristol, and the Wellcome Trust Institutional Strategic Support Fund, grant code: 204813/Z/16/Z, by The Cure Parkinson's Trust, grant code AW021 and by IXICO plc, grant code R101507-101.

Dr Jonathan de Pass and Mrs Georgina de Pass made a charitable donation to the University of Bristol (of which Jonathan is a graduate) through the Development and Alumni Relations Office to



For peer review only



UOB Confidential

support research into Parkinson's Disease. The funding pays for the salary of CM, the PhD student and Clinical Research Fellow, and she reports to the donors on her progress, but they do not have any influence on the direction of the study.

**Author Statement**

CM drafted the study protocol, worked with all contributors to improve and finalise details and continues as the study Principal Investigator. IC and AW supervise the studies and work of CM; AW is study Chief Investigator; IC is Principal of SPHERE-IRC; both have major influence on the study's direction. ET has advised on data management. KK and RoM have done pilot work and have advised on study direction. MM, FH, RyM, ET and SW have been involved in the computer science and analytics planning. JC, AH, MR and RE have given practical input on study tools and documentation. OW and HM are representatives of the Public and Patient Involvement groups which have had strong influence on study design. LR and WM have added expertise in the field to advise CM on early study design.

The study sponsor is the University of Bristol (research-governance@bristol.ac.uk). The sponsor has had no role in study design, collection, management, analysis, interpretation of the data, writing the report or the decision to submit the study for publications and will have no ultimate authority over these activities.

**Conflicts of interests**

K Kinnunen is employed by IXICO plc, who have provided some financial support of the study. The research may lead to the development of products which may be licensed to IXICO plc.

None of the other authors have any financial or other competing interests to declare.

**References**

1. Jankovic J. Parkinson's disease: clinical features and diagnosis. *J Neurol Neurosurg Psychiatry* 2008;79(4):368–76.

2. Fearnley JM, Lees AJ. Ageing and Parkinson's disease: substantia nigra regional selectivity. *Brain* 1991;114(Pt 5):2283–301.

3. Lang AE, Melamed E, Poewe W, et al. Trial designs used to study neuroprotective therapy in Parkinson's disease. *Mov Disord* 2013;28(1):86–95.

4. Goetz CG, Fahn S, Martinez-Martin P, et al. Movement Disorder Society-sponsored revision of the Unified Parkinson's Disease Rating Scale (MDS-UPDRS): Process, format, and clinimetric testing plan. *Mov Disord* 2007;22(1):41–7.

5. Goetz CG, Tilley BC, Shaftman SR, et al. Movement Disorder Society-Sponsored Revision of the Unified Parkinson's Disease Rating Scale (MDS-UPDRS): Scale presentation and clinimetric testing results. *Mov Disord* 2008;23(15):2129–70.

6. Post B, Merkus MP, de Bie RMA, et al. Unified Parkinson's disease rating scale motor examination: are ratings of nurses, residents in neurology, and movement disorders specialists interchangeable? *Mov Disord* 2005 Dec;20(12):1577–84.

7. Mcghee DJM, Ritchie CW, Zajicek JP, et al. A review of clinical trial designs used to detect a



For peer review only

For peer review only - http://bmjopen.bmj.com/site/about/guidelines.xhtml

UOB Confidential

disease-modifying effect of drug therapy in Alzheimer's disease and Parkinson's disease. *BMC Neurol* 2016;16:92.

8. Robles-García V, Corral-Bergantiños Y, Espinosa N, et al. Spatiotemporal Gait Patterns During Overt and Covert Evaluation in Patients With Parkinson´s Disease and Healthy Subjects: Is There a Hawthorne Effect? *J Appl Biomech* 2015;31(3):189–94.

9. Cubo E, Mariscal N, Solano B, Becerra V, Armesto D, Calvo S, et al. Prospective study on cost-effectiveness of home-based motor assessment in Parkinson's disease. *J Telemed Telecare* 2017;23(2):328–38.

10. Duncan RP, Earhart GM. Measuring participation in individuals with Parkinson disease: Relationships with disease severity, quality of life, and mobility. *Disabil Rehabil* 2011;33(15–16):1440–6.

11. Silva de Lima AL, Smits T, Darweesh SKL, et al. Home-based monitoring of falls using wearable sensors in Parkinson's disease. *Mov Disord* 2019;35:109–15.

12. Van Uem JMT, Isaacs T, Lewin A, et al. A viewpoint on wearable technology-enabled measurement of wellbeing and health-related quality of life in Parkinson's disease. *Journal of Parkinson's Disease* 2016;6(2):279–87.

13. Godinho C, Domingos J, Cunha G, et al. A systematic review of the characteristics and validity of monitoring technologies to assess Parkinson's disease. *J Neuroeng Rehabil* 2016;13:24.

14. Sánchez-Ferro Á, Elshehabi M, Godinho C, et al. New methods for the assessment of Parkinson's disease (2005 to 2015): A systematic review. *Mov Disord* 2016;31:1283–92.

15. Griffiths RI, Kotschet K, Arfon S, et al. Automated assessment of bradykinesia and dyskinesia in Parkinson's disease. *J Parkinsons Dis* 2012;2(1):47–55.

16. Dranca L, de Abetxuko Ruiz de Mendarozketa L, Goñi A, et al. Using Kinect to classify Parkinson's disease stages related to severity of gait impairment. *BMC Bioinformatics* 2018;19(1).

17. Artusi CA, Mishra M, Latimer P, et al. Integration of technology-based outcome measures in clinical trials of Parkinson and other neurodegenerative diseases. *Parkinsonism Relat Disord* 2018;46 Suppl 1:S53–6.

18. Espay AJ, Hausdorff JM, Sánchez-Ferro Á, et al. A roadmap for implementation of patient-centered digital outcome measures in Parkinson's disease obtained using mobile health technologies. *Mov Disord* 2019;34(5):657–63.

19. Ellis T, Cavanaugh JT, Earhart GM, et al. Which Measures of Physical Function and Motor Impairment Best Predict Quality of Life in Parkinson's Disease? *Parkinsonism Relat Disord* 2011;17(9):693-7.

20. Del Din S, Godfrey A, Mazzà C, et al. Free-living monitoring of Parkinson's disease: Lessons from the field. *Mov Disord* 2016;31(9):1293–313.

21. Woznowski P, Burrows A, Diethe T, et al. SPHERE: A Sensor Platform for Healthcare in a Residential Environment. Designing, Developing, and Facilitating Smart Cities. Cham: Springer International Publishing 2017:315–33.

22. Zhu N, Diethe T, Camplani M, et al. Bridging e-Health and the Internet of Things: The SPHERE

For peer review only



For peer review only - http://bmjopen.bmj.com/site/about/guidelines.xhtml

UOB Confidential

Project. IEEE Intell Syst [Internet]. 2015 Jul [cited 2018 Nov 15];30(4):39–46. Available from: http://ieeexplore.ieee.org/document/7156004/

23. Burrows A, Gooberman-Hill R, Coyle D. Empirically derived user attributes for the design of home healthcare technologies. *Pers Ubiquitous Comput* 2015;19(8):1233–45.

24. Burrows A, Gooberman-Hill R, Coyle D. Shared Language and the Design of Home Healthcare Technology. *Proceedings of the CHI Conference on Human Factors in Computing Systems* 2016:3584-94.

25. Grant S, Blom AW, Whitehouse MR, et al. Using home sensing technology to assess outcome and recovery after hip and knee replacement in the UK: The HEmiSPHERE study protocol. *BMJ Open* 2018;8(7):e021862.

26. Camplani M, Hannuna S, Mirmehdi M, et al. Real-time RGB-D Tracking with Depth Scaling Kernelised Correlation Filters and Occlusion Handling. *Procedings of the British Machine Vision Conference* 2015:145.1-145.11.

27. Tao L, Burghardt T, Hannuna S, et al. A comparative home activity monitoring study using visual and inertial sensors. *17th International Conference on E-health Networking, Application & Services (HealthCom)* 2015:644–7.

28. Hughes AJ, Daniel SE, Kilford L, et al. Accuracy of clinical diagnosis of idiopathic Parkinson's disease: A clinico-pathological study of 100 cases. *J Neurol Neurosurg Psychiatry* 1992;55(3):181–4.

29. Trenkwalder C, Kohnen R, Högl B, et al. Parkinson's disease sleep scale-validation of the revised version PDSS-2. *Mov Disord* 2011 Mar;26(4):644–52.

30. Martinez-Martin P, Rodriguez-Blazquez C, Abe K, et al. International study on the psychometric attributes of the Non-Motor Symptoms Scale in Parkinson disease. *Neurology* 2009;73(19):1584–91.

31. Morris S, Morris ME, Iansek R. Reliability of measurements obtained with the Timed "Up & Go" test in people with Parkinson disease. *Phys Ther* 2001 Feb;81(2):810–8.

32. Stiasny-Kolster K, Mayer G, Schäfer S, et al. The REM sleep behavior disorder screening questionnaire-A new diagnostic instrument. *Mov Disord* 2007;22(16):2386–93.

33. Marinus J, Ramaker C, van Hilten JJ, et al. Health related quality of life in Parkinson's disease: a systematic review of disease specific instruments. *J Neurol Neurosurg Psychiatry* 2002;72(2):241–8.

34. Belić M, Bobić V, Badža M, et al. Artificial intelligence for assisting diagnostics and assessment of Parkinson's disease—A review. *Clinical Neurology and Neurosurgery* 2019;184:105442.

35. Braun V, Clarke V. Using thematic analysis in psychology. *Qual Res Psychol* 2006;3(2):77–101.

36. Rosen LD, Whaling K, Carrier LM, et al. The Media and Technology Usage and Attitudes Scale: An empirical investigation. *Comput Human Behav* 2013;29(6):2501–11.

**Figure legends**

Figure 1. Flow chart detailing outline of PD SENSORS study contacts.



For peer review only

For peer review only - http://bmjopen.bmj.com/site/about/guidelines.xhtml

UOB Confidential

*PIS = Participant Information Sheet*

Figure 2. A visualisation of the day-to-day participant activities during Contact D, the SPHERE house stay.

*OFF medications = the participant with PD in practically-defined 'off' medication state which entails withholding dopaminergic medications for a period of more than 12 hours; ON medications = the participant with PD having taken their dopaminergic medications.*

For peer review only


UOB Confidential

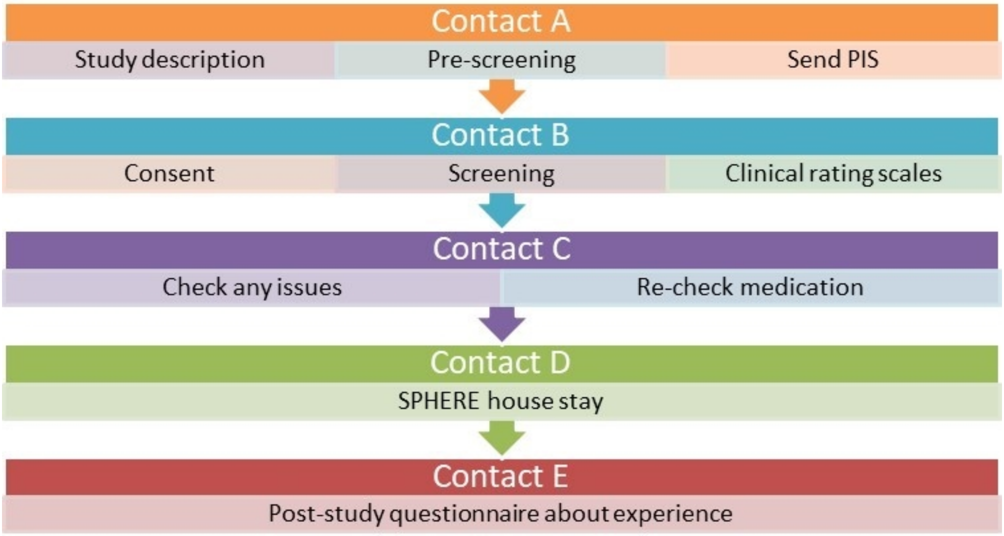


Figure 1. Flow chart detailing outline of PD SENSORS study contacts.

PIS = Participant Information Sheet

159x89mm (120 x 120 DPI)

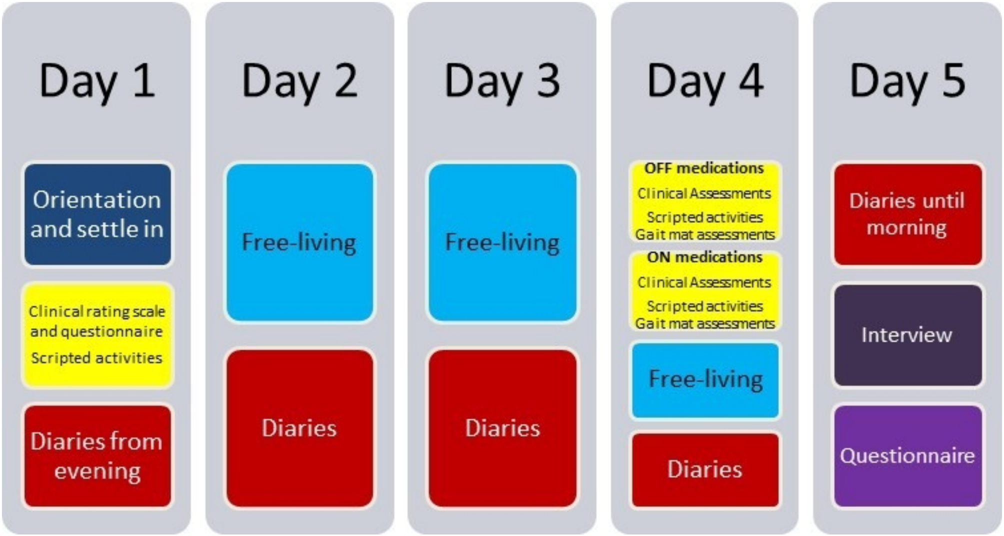


Figure 2. A visualisation of the day-to-day participant activities during Contact D, the SPHERE house stay.

OFF medications = the participant with PD in practically-defined ‘off’ medication state which entails withholding dopaminergic medications for a period of more than 12 hours; ON medications = the participant with PD having taken their dopaminergic medications.

159x84mm (120 x 120 DPI)

**Supplementary Material**

A list of some of the specific movements/actions to be evaluated in the scripted activities during Contact D

- Spreading hummus on bread
- Cutting
- Pouring
- Scooping
- Stirring
- Turning tap on and off
- Wiping surface
- Dispensing soap

For peer review only



For peer review only - http://bmjopen.bmj.com/site/about/guidelines.xhtml

# Reporting checklist for protocol of a clinical trial.

Based on the SPIRIT guidelines.

## Instructions to authors

Complete this checklist by entering the page numbers from your manuscript where readers will find each of the items listed below.

Your article may not currently address all the items on the checklist. Please modify your text to include the missing information. If you are certain that an item does not apply, please write "n/a" and provide a short explanation.

Upload your completed checklist as an extra file when you submit to a journal.

In your methods section, say that you used the SPIRITreporting guidelines, and cite them as:

Chan A-W, Tetzlaff JM, Altman DG, Laupacis A, Gøtzsche PC, Krleža-Jerić K, Hróbjartsson A, Mann H, Dickersin K, Berlin J, Doré C, Parulekar W, Summerskill W, Groves T, Schulz K, Sox H, Rockhold FW, Rennie D, Moher D. SPIRIT 2013 Statement: Defining standard protocol items for clinical trials. Ann Intern Med. 2013;158(3):200-207

| | | Reporting Item | Page Number |
|---|---|---|---|
| **Administrative information** | | | |
| Title | #1 | Descriptive title identifying the study design, population, interventions, and, if applicable, trial acronym | 1 |

For peer review only

| | | | |
|---|---|---|---|
| Trial registration | #2a | Trial identifier and registry name. If not yet registered, name of intended registry | 1 |
| Trial registration: data set | #2b | All items from the World Health Organization Trial Registration Data Set | N/A |
| Protocol version | #3 | Date and version identifier | 4 |
| Funding | #4 | Sources and types of financial, material, and other support | 14 |
| Roles and responsibilities: contributorship | #5a | Names, affiliations, and roles of protocol contributors | 14-15 |
| Roles and responsibilities: sponsor contact information | #5b | Name and contact information for the trial sponsor | 15 |
| Roles and responsibilities: sponsor and funder | #5c | Role of study sponsor and funders, if any, in study design; collection, management, analysis, and interpretation of data; writing of the report; and the decision to submit the report for publication, including whether they will have ultimate authority over any of these activities | 15 |
| Roles and responsibilities: committees | #5d | Composition, roles, and responsibilities of the coordinating centre, steering committee, endpoint adjudication committee, data management team, and other individuals or groups overseeing the trial, if applicable (see Item 21a for data | 13, 15 |

For peer review only

| | | | |
|---|---|---|---|
| | | monitoring committee) | |
| **Introduction** | | | |
| Background and rationale | #6a | Description of research question and justification for undertaking the trial, including summary of relevant studies (published and unpublished) examining benefits and harms for each intervention | 3-5 |
| Background and rationale: choice of comparators | #6b | Explanation for choice of comparators | 11 |
| Objectives | #7 | Specific objectives or hypotheses | 5 |
| Trial design | #8 | Description of trial design including type of trial (eg, parallel group, crossover, factorial, single group), allocation ratio, and framework (eg, superiority, equivalence, non-inferiority, exploratory) | 5 |
| **Methods: Participants, interventions, and outcomes** | | | |
| Study setting | #9 | Description of study settings (eg, community clinic, academic hospital) and list of countries where data will be collected. Reference to where list of study sites can be obtained | 5 |
| Eligibility criteria | #10 | Inclusion and exclusion criteria for participants. If applicable, | 6 |

For peer review only

| | | | |
|---|---|---|---|
| | | eligibility criteria for study centres and individuals who will perform the interventions (eg, surgeons, psychotherapists) | |
| Interventions: description | #11a | Interventions for each group with sufficient detail to allow replication, including how and when they will be administered | 6-9 |
| Interventions: modifications | #11b | Criteria for discontinuing or modifying allocated interventions for a given trial participant (eg, drug dose change in response to harms, participant request, or improving / worsening disease) | N/A |
| Interventions: adherance | #11c | Strategies to improve adherence to intervention protocols, and any procedures for monitoring adherence (eg, drug tablet return; laboratory tests) | N/A |
| Interventions: concomitant care | #11d | Relevant concomitant care and interventions that are permitted or prohibited during the trial | 6 |
| Outcomes | #12 | Primary, secondary, and other outcomes, including the specific measurement variable (eg, systolic blood pressure), analysis metric (eg, change from baseline, final value, time to event), method of aggregation (eg, median, proportion), and time point for each outcome. Explanation of the clinical relevance of chosen efficacy and harm outcomes is strongly recommended | 5, 7, 11-13 |
| Participant timeline | #13 | Time schedule of enrolment, interventions (including any run-ins and washouts), assessments, and visits for participants. A schematic diagram is highly recommended (see Figure) | 7 |

For peer review only

| | | | |
|---|---|---|---|
| Sample size | #14 | Estimated number of participants needed to achieve study objectives and how it was determined, including clinical and statistical assumptions supporting any sample size calculations | 11 |
| Recruitment | #15 | Strategies for achieving adequate participant enrolment to reach target sample size | 6 |
| **Methods: Assignment of interventions (for controlled trials)** | | | |
| Allocation: sequence generation | #16a | Method of generating the allocation sequence (eg, computer-generated random numbers), and list of any factors for stratification. To reduce predictability of a random sequence, details of any planned restriction (eg, blocking) should be provided in a separate document that is unavailable to those who enrol participants or assign interventions | N/A |
| Allocation concealment mechanism | #16b | Mechanism of implementing the allocation sequence (eg, central telephone; sequentially numbered, opaque, sealed envelopes), describing any steps to conceal the sequence until interventions are assigned | N/A |
| Allocation: implementation | #16c | Who will generate the allocation sequence, who will enrol participants, and who will assign participants to interventions | N/A |
| Blinding | #17a | Who will be blinded after assignment to interventions (eg, trial | N/A |

For peer review only

| | | | |
|---|---|---|---|
| (masking) | | participants, care providers, outcome assessors, data analysts), and how | |
| Blinding (masking): emergency unblinding | #17b | If blinded, circumstances under which unblinding is permissible, and procedure for revealing a participant's allocated intervention during the trial | N/A |
| **Methods: Data collection, management, and analysis** | | | |
| Data collection plan | #18a | Plans for assessment and collection of outcome, baseline, and other trial data, including any related processes to promote data quality (eg, duplicate measurements, training of assessors) and a description of study instruments (eg, questionnaires, laboratory tests) along with their reliability and validity, if known. Reference to where data collection forms can be found, if not in the protocol | 7-10 |
| Data collection plan: retention | #18b | Plans to promote participant retention and complete follow-up, including list of any outcome data to be collected for participants who discontinue or deviate from intervention protocols | 13 |
| Data management | #19 | Plans for data entry, coding, security, and storage, including any related processes to promote data quality (eg, double data | 13 |

For peer review only

| | | | |
|---|---|---|---|
| | | entry; range checks for data values). Reference to where details of data management procedures can be found, if not in the protocol | |
| Statistics: outcomes | #20a | Statistical methods for analysing primary and secondary outcomes. Reference to where other details of the statistical analysis plan can be found, if not in the protocol | 11-13 |
| Statistics: additional analyses | #20b | Methods for any additional analyses (eg, subgroup and adjusted analyses) | 11-13 |
| Statistics: analysis population and missing data | #20c | Definition of analysis population relating to protocol non-adherence (eg, as randomised analysis), and any statistical methods to handle missing data (eg, multiple imputation) | N/A |
| **Methods: Monitoring** | | | |
| Data monitoring: formal committee | #21a | Composition of data monitoring committee (DMC); summary of its role and reporting structure; statement of whether it is independent from the sponsor and competing interests; and reference to where further details about its charter can be found, if not in the protocol. Alternatively, an explanation of why a DMC is not needed | 12 |
| Data monitoring: interim analysis | #21b | Description of any interim analyses and stopping guidelines, including who will have access to these interim results and | N/A |

For peer review only

| | | | |
|---|---|---|---|
| | | make the final decision to terminate the trial | |
| Harms | #22 | Plans for collecting, assessing, reporting, and managing solicited and spontaneously reported adverse events and other unintended effects of trial interventions or trial conduct | 9-10 |
| Auditing | #23 | Frequency and procedures for auditing trial conduct, if any, and whether the process will be independent from investigators and the sponsor | 13 |
| **Ethics and dissemination** | | | |
| Research ethics approval | #24 | Plans for seeking research ethics committee / institutional review board (REC / IRB) approval | 6 |
| Protocol amendments | #25 | Plans for communicating important protocol modifications (eg, changes to eligibility criteria, outcomes, analyses) to relevant parties (eg, investigators, REC / IRBs, trial participants, trial registries, journals, regulators) | 11 |
| Consent or assent | #26a | Who will obtain informed consent or assent from potential trial participants or authorised surrogates, and how (see Item 32) | 8 |
| Consent or assent: ancillary studies | #26b | Additional consent provisions for collection and use of participant data and biological specimens in ancillary studies, if applicable | N/A |
| Confidentiality | #27 | How personal information about potential and enrolled participants will be collected, shared, and maintained in order | 14 |

For peer review only

| | | | |
|---|---|---|---|
| | | to protect confidentiality before, during, and after the trial | |
| Declaration of interests | #28 | Financial and other competing interests for principal investigators for the overall trial and each study site | 14 |
| Data access | #29 | Statement of who will have access to the final trial dataset, and disclosure of contractual agreements that limit such access for investigators | 13 |
| Ancillary and post trial care | #30 | Provisions, if any, for ancillary and post-trial care, and for compensation to those who suffer harm from trial participation | N/A |
| Dissemination policy: trial results | #31a | Plans for investigators and sponsor to communicate trial results to participants, healthcare professionals, the public, and other relevant groups (eg, via publication, reporting in results databases, or other data sharing arrangements), including any publication restrictions | 13-14 |
| Dissemination policy: authorship | #31b | Authorship eligibility guidelines and any intended use of professional writers | N/A |
| Dissemination policy: reproducible research | #31c | Plans, if any, for granting public access to the full protocol, participant-level dataset, and statistical code | 13 |
| **Appendices** | | | |
| Informed consent materials | #32 | Model consent form and other related documentation given to participants and authorised surrogates | Appendix |

For peer review only

| | | | |
|---|---|---|---|
| Biological specimens | #33 | Plans for collection, laboratory evaluation, and storage of biological specimens for genetic or molecular analysis in the current trial and for future use in ancillary studies, if applicable | N/A |

None The SPIRIT checklist is distributed under the terms of the Creative Commons Attribution License CC-BY-ND 3.0. This checklist can be completed online using https://www.goodreports.org/, a tool made by the EQUATOR Network in collaboration with Penelope.ai

For peer review only

BMJ Open

# Protocol for PD SENSORS: Parkinson's Disease Symptom Evaluation in a Naturalistic Setting producing Outcomes measuRes using SPHERE technology. An observational feasibility study of multi-modal multi-sensor technology to measure symptoms and activities of daily living in Parkinson's disease.



| | |
|---|---|
| Journal: | *BMJ Open* |
| Manuscript ID | bmjopen-2020-041303.R2 |
| Article Type: | Protocol |
| Date Submitted by the Author: | 16-Oct-2020 |





| | |
|---|---|
| Complete List of Authors: | Morgan, Catherine; University of Bristol Medical School, Translational Health Sciences; North Bristol NHS Trust, Movement Disorders Group,<br>Craddock, Ian ; University of Bristol, School of Computer Science, Electrical and Electronic Engineering, and Engineering Mathematics, Faculty of Engineering<br>Tonkin, Emma; University of Bristol, School of Computer Science, Electrical and Electronic Engineering, and Engineering Mathematics, Faculty of Engineering<br>Kinnunen, Kirsi; IXICO plc<br>McNaney, Roisin; University of Bristol, School of Computer Science, Electrical and Electronic Engineering, and Engineering Mathematics, Faculty of Engineering<br>Whitehouse, Sam; University of Bristol, School of Computer Science, Electrical and Electronic Engineering, and Engineering Mathematics, Faculty of Engineering<br>Mirmehdi, Majid; University of Bristol, School of Computer Science, Electrical and Electronic Engineering, and Engineering Mathematics, Faculty of Engineering<br>Heidarivincheh, Farnoosh; University of Bristol, School of Computer Science, Electrical and Electronic Engineering, and Engineering Mathematics, Faculty of Engineering<br>McConville, Ryan; University of Bristol, School of Computer Science, Electrical and Electronic Engineering, and Engineering Mathematics, Faculty of Engineering<br>Carey, Julia; University of Bristol, School of Computer Science, Electrical and Electronic Engineering, and Engineering Mathematics, Faculty of Engineering<br>Horne, Alison; University of Bristol Medical School<br>Rolinski, Michal; University of Bristol Medical School, Translational Health Sciences; North Bristol NHS Trust, Movement Disorders Group,<br>Rochester, Lynn; Newcastle University, Institute of Neuroscience; Newcastle Upon Tyne Hospitals NHS Foundation Trust<br>Maetzler, Walter; University Medical Center Schleswig-Holstein Campus Kiel, Department of Neurology<br>Matthews, Helen; Cure Parkinson's Trust |

| | |
|---|---|
| | Watson, Oliver; Bristol Health Partners<br>Eardley, Rachel; University of Bristol, School of Computer Science, Electrical and Electronic Engineering, and Engineering Mathematics, Faculty of Engineering<br>Whone, Alan; University of Bristol , School of Translational Health Sciences ; North Bristol NHS Trust, Movement Disorders Group |
| <b>Primary Subject Heading</b>: | Neurology |
| Secondary Subject Heading: | Research methods |
| Keywords: | Parkinson-s disease < NEUROLOGY, Information technology < BIOTECHNOLOGY & BIOINFORMATICS, QUALITATIVE RESEARCH, STATISTICS & RESEARCH METHODS |
| | |

SCHOLARONE™ Manuscripts

*I, the Submitting Author has the right to grant and does grant on behalf of all authors of the Work (as defined in the below author licence), an exclusive licence and/or a non-exclusive licence for contributions from authors who are: i) UK Crown employees; ii) where BMJ has agreed a CC-BY licence shall apply, and/or iii) in accordance with the terms applicable for US Federal Government officers or employees acting as part of their official duties; on a worldwide, perpetual, irrevocable, royalty-free basis to BMJ Publishing Group Ltd ("BMJ") its licensees and where the relevant Journal is co-owned by BMJ to the co-owners of the Journal, to publish the Work in this journal and any other BMJ products and to exploit all rights, as set out in our licence.*

*The Submitting Author accepts and understands that any supply made under these terms is made by BMJ to the Submitting Author unless you are acting as an employee on behalf of your employer or a postgraduate student of an affiliated institution which is paying any applicable article publishing charge ("APC") for Open Access articles. Where the Submitting Author wishes to make the Work available on an Open Access basis (and intends to pay the relevant APC), the terms of reuse of such Open Access shall be governed by a Creative Commons licence – details of these licences and which Creative Commons licence will apply to this Work are set out in our licence referred to above.*

*Other than as permitted in any relevant BMJ Author's Self Archiving Policies, I confirm this Work has not been accepted for publication elsewhere, is not being considered for publication elsewhere and does not duplicate material already published. I confirm all authors consent to publication of this Work and authorise the granting of this licence.*

For peer review only

# Protocol for PD SENSORS: Parkinson's Disease Symptom Evaluation in a Naturalistic Setting producing Outcomes measuRes using SPHERE technology. An observational feasibility study of multi-modal multi-sensor technology to measure symptoms and activities of daily living in Parkinson's disease.

Authors: Catherine Morgan [a,c,*] , Ian Craddock [b], Emma Tonkin [b], Kirsi M Kinnunen [d], Roisin McNaney [b], Sam Whitehouse [b], Majid Mirmehdi [b], Farnoosh Heidarivincheh [b], Ryan McConville [b], Julia Carey [b], Alison Horne [e], Michal Rolinski [a,c], Lynn Rochester [f,g], Walter Maetzler [h], Helen Matthews [i], Oliver Watson [j], Rachel Eardley [b], Alan L Whone [a,c]

[a] University of Bristol Medical School, Translational Health Sciences, Bristol, UK

[b] University of Bristol, School of Computer Science, Electrical and Electronic Engineering, and Engineering Mathematics, Faculty of Engineering, Bristol, UK

[c] North Bristol NHS Trust, Movement Disorders Group, Westbury on Trym, Avon, UK

[d] IXICO plc, London, UK

[e] University of Bristol Medical School, Bristol, UK

[f] Newcastle University, Institute of Neuroscience, Newcastle, UK

[g] Newcastle Upon Tyne Hospitals NHS Foundation Trust, Newcastle Upon Tyne, UK

[h] University Medical Center Schleswig-Holstein Campus Kiel, Department of Neurology, Kiel, Schleswig-Holstein, DE

[i] Cure Parkinson's Trust, London, UK

[j] Bristol Health Partners, Bristol, UK

**Emails of authors**: Catherine.morgan@bristol.ac.uk; ian.craddock@bristol.ac.uk; e.l.tonkin@bristol.ac.uk; kirsi.kinnunen@ixico.com; r.mcnaney@bristol.ac.uk; sw12690@bristol.ac.uk; m.mirmehdi@bristol.ac.uk; farnoosh.heidarivincheh@bristol.ac.uk; ryan.mcconville@bristol.ac.uk; Julia.carey@bristol.ac.uk; alison.horne@bristol.ac.uk; michal.rolinski@bristol.ac.uk; lynn.rochester@newcastle.ac.uk; w.maetzler@neurologie.uni-kiel.de; helen@cureparkinsons.org.uk; oliver.watson@bristol.ac.uk; Rachel.eardley@bristol.ac.uk; alan.whone@bristol.ac.uk

**Correspondence to:** Dr Catherine Morgan; Catherine.morgan@bristol.ac.uk; Movement Disorders Group, Bristol Brain Centre, Southmead Hospital, North Bristol National Health Service Trust, Bristol, UK; +44 7970 657958

**Registration:** University of Bristol Research Registration Database, reference 2018-4247

**Keywords:** Parkinson's disease; Information technology; Qualitative research; Statistics and research methods



For peer review only - http://bmjopen.bmj.com/site/about/guidelines.xhtml

UOB Confidential

**Word count:** 4991

## Abstract

Introduction: The impact of disease-modifying agents on disease progression in Parkinson's disease is largely assessed in clinical trials using clinical rating scales. These scales have drawbacks in terms of their ability to capture the fluctuating nature of symptoms while living in a naturalistic environment. The SPHERE (Sensor Platform for HEalthcare in a Residential Environment) project has designed a multi-sensor platform with multimodal devices designed to allow continuous, relatively inexpensive, unobtrusive sensing of motor, non-motor and activities of daily living metrics in a home or a home-like environment. The aim of this study is to evaluate how the SPHERE technology can measure aspects of Parkinson's disease.

Methods and Analysis: This is a small-scale feasibility and acceptability study during which 12 pairs of participants (comprising a person with Parkinson's and a healthy control participant) will stay and live freely for 5 days in a home-like environment embedded with SPHERE technology including environmental, appliance monitoring, wrist-worn accelerometry and silhouette video sensors. These data will be collected alongside clinical rating scales, participant diary entries and expert clinician annotations of colour video images. Machine learning will be used to look for a signal to discriminate between Parkinson's disease and control, and between Parkinson's disease symptoms 'on' or 'off' medications. Additional outcome measures including bradykinesia, activity level, sleep parameters and some activities of daily living will be explored. Acceptability of the technology will be evaluated qualitatively using semi-structured interviews.

Ethics and Dissemination: Ethical approval has been given to commence this study; the results will be disseminated as widely as appropriate.

For peer review only

Strengths of this study:

- The location for testing (SPHERE house) allows free living whilst providing some ground truth through colour video capture of participants' activities for clinician annotation.
- Due to the multi-sensor data collection platform within the SPHERE house, the performance of machine learning models which are based on single or multiple modalities could be assessed.
- The qualitative tools will add the important dimension of the person with Parkinson's perspective on this technology.

Limitations of this study:

- The small number of participants means that results generated from this study will not be generalizable to the wider population of people with PD.
- The feasibility nature of PD SENSORS means that cost effectiveness will not be explored at this stage.

## Introduction

Parkinson's disease (PD) is the second most common neurodegenerative disease in the UK, affecting more than 127,000 people. It is a chronic, progressive, disabling disease which is characterised by a



For peer review only - http://bmjopen.bmj.com/site/about/guidelines.xhtml

UOB Confidential

large variety of possible motor symptoms (including bradykinesia, rigidity and tremor) and non-motor symptoms (including sleep disturbance, cognitive impairment and pain) (1). It is estimated that by the time of diagnosis with PD most patients have already lost around 50% of their dopaminergic neurones (2), therefore attempts to modify the disease should be aimed at pre-clinical or early-stage disease states.

The search for a treatment which could modify PD, a so-called disease-modifying therapy or 'DMT', has thus far proven fruitless with no DMT reaching market authorisation. This is despite multiple potential therapies having been tested in randomised, double-blind clinical trials (3).

Clinical trials testing DMTs have often used a clinical rating scale to quantify symptoms and monitor response to treatment as a primary outcome measure (3). The gold-standard rating scale is the Movement Disorder Society-sponsored revision of the Unified Parkinson's Disease Rating Scale (MDS-UPDRS) (4). This is a validated (5) tool which evaluates a comprehensive spectrum of symptoms and activities. However it is also a subjective (6) and non-linear (7) tool which exposes the study participant to the risk of observer bias and the 'Hawthorne Effect' (8). It is used episodically, giving a snapshot of a person with PD, which may not capture the day-to-day and hour-to-hour fluctuations of this complex disease. The testing location is often an artificial environment like a clinic or laboratory. This can increase costs (9) and impair the ability of studies to appraise important activities of daily living (ADLs) such as engagement in social activities (10), rare events such as falls (11) and other metrics which affect wellbeing and quality of life such as sleep (12).

Development of technologies for the measurement of various aspects of PD have been evolving over the past 15 years (13). Particularly motor symptoms, including gait and bradykinesia, are amenable to measurement by technology (14) continuously and over long time periods. For example, the Parkinson's KinetiGraph wearable movement recording device (developed by Global Kinetics) can measure tremor, bradykinesia and dyskinesia(15) and the Microsoft Kinect motion sensor can classify PD stages relating to gait impairment (16). However, these tools are still only partially integrated into clinical trials, with most continuing to use periodic clinical rating scale assessments in the non-ecological setting as primary or secondary end-points (17). There are excellent initiatives which are guiding this field forward, for example a 'roadmap' for implementation of digital outcome measures using technologies (18), which are tackling the important questions surrounding regulatory approvals and collaboration with industry.

Lacunae in the literature about free-living technology-assisted outcomes in PD include the measurement of non-motor outcomes (14) such as constipation and activities of daily living. The frequent use of wearable devices which are attached to specific body parts means that the information gathered is restricted to the movement in that part of the body; this may be inadequate fully to understand the context in which a measurement is taken and may result in low correlations with measures of functional disability and/or quality of life (19). In addition, it can be challenging to establish and operationalise the 'ground truth' for benchmarking novel measurements. Reviews of technology-assisted outcome measures in a free-living environment have highlighted that to date there is no fully-validated system which evaluates clinical features of PD in a naturalistic/ecologically valid (i.e. home or home-like) environment (20).

<u>Sensor Platform for HEalthcare in a Residential Environment - SPHERE</u>



For peer review only - http://bmjopen.bmj.com/site/about/guidelines.xhtml

UOB Confidential

For peer review only

The long-standing SPHERE project from the University of Bristol (SPHERE: Sensor Platform for HEalthcare in a Residential Environment) has led to the design of a multi-sensor platform with multimodal devices designed to allow continuous, relatively inexpensive, unobtrusive sensing of motor, non-motor and ADL metrics in a home or a home-like environment (21,22).

The SPHERE sensor platform is embedded into a test-bed location called the 'SPHERE house'. This is a 2-bedroom, 2-storey property with kitchen, living room, dining room, bathroom, and toilet.

Within the SPHERE house there are a number of sensors which include: wearable devices with accelerometers (in-house developed); environmental/ambient sensors which measure temperature, humidity and light levels; RGB-D ('red green blue – depth', referring to the three colour planes that together can generate the colour of a pixel in an image, in addition to the recording of distances to surfaces using infra-red light) camera sensors that generate silhouettes of participants and bounding boxes that, when linked with the SPHERE wearable, let the system know who the participant is; appliance sensors which are able to tell when and how frequently an appliance is switched on and off; PIR (Passive InfraRed) sensors which detect motion in each room.

The SPHERE sensor platform has undergone extensive testing with healthy volunteers, being deployed to 50 homes over a year from January 2017. High-quality qualitative work has been conducted looking at the design and acceptability of the SPHERE sensors and technology in a person's life (23,24). In addition, the SPHERE platform is being utilised in 3 clinical projects which are at different stages of completion, looking at outcomes around cardiac valve replacement, hip and knee surgery (25) and in dementia/mild cognitive impairment.

The multimodal multi-sensor design of the SPHERE system (22) enables a richer and more fine-grained picture of free-living symptoms and activities to be collected. For example, the combined use of the wearable devices with accelerometers, alongside the video and environmental sensors, enables machine learning algorithms to opportunistically and continuously fuse data to build a time series of activities of daily living for each person in the home (26,27). This offers an exciting opportunity to conduct free-living evaluation of people with PD.

Parkinson's Disease Symptom Evaluation in a Naturalistic Setting producing Outcomes measuRes using SPHERE technology – PD SENSORS

Given the above gaps in knowledge, and appreciating the SPHERE system's capability, it is believed that SPHERE technology could be exploited for filling some of the gaps in the current use of technology to measure outcomes in PD (e.g. for non-motor symptoms and ADLs). SPHERE technology has the potential to complement and enhance the existing clinical rating scales used to evaluate Parkinson's disease. PD SENSORS is a feasibility and acceptability study using the SPHERE platform to develop novel outcome measures in PD. It is designed with the intent that it will inform a future larger scale and longer-term investigation, in which SPHERE technology will be deployed to multiple homes of people with PD (protocol version 1.3; August 2020). This is a truly collaborative study with not only strong interdisciplinary collaboration between computer scientists and movement disorders clinicians but also industry involvement. The university academic team are working with a UK-based imaging and digital biomarker analysis company, IXICO, that contributes clinical and data science expertise and is providing additional wearable devices from Axivity (AX3)



For peer review only - http://bmjopen.bmj.com/site/about/guidelines.xhtml

UOB Confidential

and Activinsights (GENEActiv Original) and gait mat technology from ProtoKinetics (Zeno instrumented walkway), to further enrich the dataset.

**Aims**

Primary aims

To understand how the raw sensor data obtained from the SPHERE system can be processed to translate into meaningful data for the clinical scientist on metrics of:

- Total body movement
- Bradykinesia
- Sleep quantity and quality
- Urinary dysfunction
- Meal preparation/consumption
- Cleaning tasks
- Getting out of a chair
- Activity level (ambulatory activity and sedentary behaviour)
- Room to room transfers and time spent in each room

Secondary aims

1. To evaluate the acceptability of the continuous use of multiple and varied SPHERE sensors over a 5-day period in a home-like setting for people with Parkinson's disease and to explore the participants' experience of such a study
2. To explore the use of home sensing as a means to discriminate between participants with PD and healthy control participants
3. To assess the criterion validity of the technology-assisted outcomes by mapping the sensor data outcomes to the gold-standard clinical rating scale (MDS-UPDRS) and patient-reported outcome measure (PDQ-39) when participants with PD are in both 'on' and 'off' medication states

**Methods**

Design

A small-scale feasibility and acceptability study.

Setting and participants

The setting will be the SPHERE house: a customised 2-bedroom residential house in Bristol, UK, with multiple embedded sensors and data collection tools.

12 pairs of participants comprising a person with PD and a control participant (likely to be a spouse, other family member, friend, or carer) will be recruited to stay in the SPHERE house for 5 consecutive days.

Participants with PD will be eligible to enter the study if they meet certain inclusion criteria, including:



For peer review only - http://bmjopen.bmj.com/site/about/guidelines.xhtml

UOB Confidential

For peer review only

- Diagnosis of idiopathic Parkinson's disease according to UK Brain Bank Criteria (28);
- Age over 18
- Modified Hoehn and Yahr Scale score of 3 or less in 'off' state (i.e. when they withhold dopaminergic medications)

Inclusion criteria for control participants includes:

- No history of Parkinson's disease, REM (rapid eye movement) sleep behaviour disorder, dementia, or other neurodegenerative/significant musculoskeletal condition

The study's exclusion criteria include:

- Montreal Cognitive Assessment Score < 26
- Current active depressive symptoms; Beck Depression Inventory II score >19
- Use of walking aids whilst inside a house to aid mobility either 'on' or 'off' medications

Exclusion criteria were largely chosen to reduce the potential impact on sensor data of other factors, such as mobility aids altering accelerometry data making it difficult to evaluate bradykinesia.

Participants will be recruited through movement disorders specialist or general neurology outpatient clinics in North Bristol NHS Trust and through posters in the outpatient department of North Bristol NHS Trust, The Cure Parkinson's Trust, a local Patient and Public Involvement Group or by word-of-mouth.

The medical PD care for those study participants with the disease will continue unchanged.

The study will be conducted in accordance with the Declaration of Helsinki and Good Clinical Practice Guidelines. Written informed consent will be obtained from all study participants before any study-related procedures are performed.

Full approval from the National Health Service (NHS) Wales Research Ethics Committee 6 was granted on 17$^{th}$ December 2019, and Health Research Authority and Health and Care Research Wales approval confirmed on 14$^{th}$ January 2020.

Due to delays relating to the COVID-19 pandemic, active study data gathering is planned to commence in October 2020 and, if uninterrupted further, should be complete within 12 months.

<u>Sample size</u>

This sample size (24) was chosen as a balance between gathering enough data from the SPHERE sensors to create excellent quality algorithms (in consultation with our Machine Learning team) and the need to pragmatically take into account the nature of this exploratory study which is developing analytics to use in deployment to people's own homes in a future study. No conventional sample size calculation has been undertaken since we have no clear effect estimates to use in any such calculation; we view this more as a pilot and a proof of concept study. The comparator of the healthy volunteer control participant was chosen in order to make the study more sociable and therefore hopefully enjoyable to the person with PD, alongside also providing study data from people without PD so that conclusions could be drawn about how to identify and then measure aspects of PD.



For peer review only - http://bmjopen.bmj.com/site/about/guidelines.xhtml

UOB Confidential

Overview of study contacts

PD SENSORS is split into 5 study ‘contacts’ (stages at which information is transmitted to or gathered from the study participants). A flow chart in Figure 1, below, illustrates the main aims of each study contact.

At each study contact, specific information is gathered from the participants by the research team. Table 1 details the tools to be used during the longer study contacts, Contacts B and D, with the participants. The items in italics in this table apply only to the participant with PD and not to the control participant.

Table 1. Baseline and follow-up data from Contacts B and D

| **Time Point** | **Outcomes Measured** |
|---|---|
| Contact B | Further demographic information including date of birth and *date of diagnosis of PD*<br>Full screening questions according to inclusion and exclusion criteria<br>*Medication regime and calculation of Levodopa equivalent daily dose*<br>*H&Y score*<br>MOCA<br>BDI II, AS, ESS<br>Timed Up and Go Test<br>*NMSS*<br>*PDSS*<br>*RBD-SQ* |
| Contact D (the SPHERE house stay) | *MDS-UPDRS (the motor sub-scale will be performed on multiple occasions, once while ‘off’ medications)*<br>*PDQ-39*<br>Symptom and activity diary<br>Sleep diary<br>Bladder diary<br>*Medication-taking record*<br>Sensor data from scripted activities<br>Sensor data from free-living<br>Sensor data from wearable devices provided through IXICO<br>Annotations of colour video dataset<br>Gait mat assessments<br>Interview with questions relating to experience of living with SPHERE technology |

*AS = Apathy Scale; BDI II = Beck’s Depression Inventory II; ESS = Epworth Sleepiness Scale; H&Y = Hoehn and Yahr; MDS-UPDRS = Movement Disorder Society sponsored revision of the Unified Parkinson’s Disease Rating Scale; MOCA = Montreal Cognitive Assessment; NMSS = Non-Motor Symptoms Scale for Parkinson’s disease; PDQ-39 = Parkinson’s Disease Questionnaire-39; PDSS-2 = Parkinson’s Disease Sleep Scale-2; RBD-SQ = REM sleep Behaviour Disorder Screening Questionnaire*

Clinical Rating Scales

Several clinical rating scales will be completed as described at Contact B. This is with the intent to gain a thorough understanding of the nature of the symptoms experienced by the participants with



For peer review only - http://bmjopen.bmj.com/site/about/guidelines.xhtml

UOB Confidential

PD, and to be able to therefore make better sense of the sensor data produced from the SPHERE house stay. The Parkinson's Disease Sleep Scale-2 is a 15-point visual analogue scale that has been validated in PD (29) which allows patients with PD to self-rate and quantify the profile of nocturnal disturbances and sleep disruption being experienced. The Non-Motor Symptoms Scale (NMSS) is a 30-question scale which assesses severity and frequency with which a patient with PD experiences a variety of non-movement related symptoms, such as urinary dysfunction, mood/cognitive symptoms and gastrointestinal tract-associated symptoms (30). The Timed Up and Go (TUG) test (31) is a simple, quick and widely-used clinical performance tool which assesses lower extremity function, mobility and falls risk. The REM sleep Behaviour Disorder Screening Questionnaire (RBD-SQ) is a useful screening tool due to its relatively high sensitivity for REM sleep behaviour disorder (32), which affects a significant proportion of patients with PD and may change the interpretation of the sensor data overnight.

During Contact D, the research team will conduct the Movement Disorder Society sponsored revision of the Unified Parkinson's Disease Rating Scale (MDS-UPDRS) (5) with the participant with Parkinson's. This clinical rating scale has subscales evaluating motor experiences of daily living (part I), non-motor experiences of daily living (part II), motor examination (part III) and motor complications (part IV). The PDQ-39, also to be used in Contact D, is a self-completed patient-reported outcome measure designed to address aspects of functioning and well-being for those affected by Parkinson's disease (33).

<u>Schedule of Contact D: the stay in the SPHERE house</u>

The pair of participants will stay in the house together for five days. The duration of stay was chosen in consultation with people with PD and their acquaintances who took part in pre-study focus groups. During the time they are in the house, the participants will be encouraged to continue living freely and undertake their regular activities of daily living as normally as possible.

Figure 2 shows a day-by-day plan for Contact D for the pair of participants. The figure illustrates that the stay in the SPHERE house for each pair of participants will also involve:

- Researchers will visit the house on two occasions, Day 1 and Day 4, to conduct scripted activities and clinical rating scales with participants.
- During Days 2 and 3 the participants will live freely with no researcher visits.

Important elements during the 5-day stay are:

- The participant with PD will withhold their dopaminergic medications for >12 hours so that they are in the practically-defined 'off' medication state. During this time 'off' medications (on Day 4), some assessments will be undertaken including the MDS-UPDRS III (motor subsection). These assessments will then be repeated after the participant with PD restarts their medications that day.
- Specific food preparation and cleaning/hygiene scripted activities, will be witnessed and annotated by a study team member during their planned visits to the house. The participant with PD will undertake these activities on Day 1 and twice on Day 4, first 'off' then 'on' medications, whilst the control participant will do the activities once each on Day 1 and Day 4. More detail about these activities is shown in the supplementary material.



For peer review only - http://bmjopen.bmj.com/site/about/guidelines.xhtml

UOB Confidential

- Continuous sensor data will be captured throughout the 5-day stay from the following SPHERE sensors: cameras (that provide silhouettes and bounding boxes), appliance sensors, environment/ambient sensors, PIR sensors.
- Wearable devices: in addition to the SPHERE wearable device on each wrist recording continuous accelerometry, an extra IXICO-provided wearable will be worn on each wrist throughout the 5-day stay. During the clinical assessments and scripted activities on day 4 one additional wearable accelerometer will be placed around the trunk to locate in the lower back (lumbar) area and another one on a lower limb (shin).

Gait mat assessments

During the 'off' and 'on' medication testing session on Day 4, the participants will undertake clinical assessments including an evaluation of gait (normal pace, slow pace and fast pace) on the Zeno Walkway, a gait mat embedded with sensors that is placed in the entrance area of SPHERE house where these assessments are conducted. The item is administered according to the standard instructions and the gait mat protocol. All gait mat protocols for these assessments have been developed by ProtoKinetics, the Zeno walkway manufacturers.

Colour video capture

In communal areas of the SPHERE house (i.e. not in bedrooms or bathrooms) cameras will be enabled to produce RGB data for the purposes of allowing more accurate and relevant evaluation of the participants' movements. Short periods of these data, collected both while participants are free-living and during clinical assessments, will be used to provide a labelled dataset for the analytic work-up of total body movement using annotations of these videos relating to the participants' activities, location, and aspects of their Parkinson's disease symptomatology.

All work during the events and circumstances relating to severe acute respiratory syndrome coronavirus (SARS-COV-2)/Coronavirus-19 (COVID-19) will be subject to risk assessment and modification to ensure the study is safe for participants and researchers.

**Data Analysis and Management**

Statistical data analysis approaches

The purpose of this preliminary and exploratory study is to explore the best way to quantify PD using the in-house technology, therefore the exact nature of the analytic approach(es) is not available at this stage. Study researchers and the study statistician will work collaboratively with the clinicians and SPHERE machine learning team to determine how the SPHERE data can be transformed into usable form. The research team will then aim to ascertain if this data can give us a signal to discriminate between participants with PD and control participants and whether it can give us a signal to discriminate between when a participant with PD is 'on' or 'off' medications. We will compare it to the data produced by the gold-standard clinical rating scales and patient-reported outcome measures. Machine learning (ML) will play a key role in analysing the data.

Existing works have proposed methods based on ML for PD diagnosis and monitoring (please refer to (34) for a detailed review). Using various types of data, such as videos, Inertial Measurement Unit (IMU) and smart-phone or wearable accelerometer data, they model the presence and/or



For peer review only - http://bmjopen.bmj.com/site/about/guidelines.xhtml

UOB Confidential

For peer review only

severity of PD via ML algorithms such as Artificial Neural Networks (ANN), Hidden Markov Models (HMM), Support Vector Machines (SVM) and Random Forests (RF). We also aim to use the data collected from the SPHERE house, which is a multi-sensor platform, with various ML techniques to assess the reliability of an automatic learning approach for modelling and analysing PD. As mentioned before, the data that will be utilised to learn from includes the output of wearable accelerometer, RGB-D cameras with silhouette extraction, PIR and environmental sensors.

To achieve such analysis, multiple potential approaches could be considered for future work, some of which are presented in the following:

PD vs non-PD recognition **–** Given the input data corresponding to different time scales, such as seconds, minutes, or hours, ML-based model could be trained to discriminate between PD and non-PD (control) subjects. Such recognition could be performed as binary classification or as a probability estimation per category. Further, the predictions corresponding to different durations could be compared to determine the one by which the most reliable recognition is achieved.

PD severity estimation **–** Based on different rating scales such as the MDS-UPDRS or H&Y score, an ML-based approach could learn to estimate the severity of the disease among the PD patients. The ground-truth scores are provided by the clinicians during both 'on' and 'off' medication periods. Then, a model could be trained to regress predictions using the ground-truth scores.

Fine-grained analysis of PD symptoms **–** In addition to PD recognition and severity estimation, an ML-based model could be trained to detect different PD symptoms, such as bradykinesia or tremor. Also, a finer-grained analysis of each symptom could be investigated. For example, a model could learn to detect peaks or changes during tremor.

Activity recognition **–** The learning could be extended towards recognising the category of the activities which are performed by the subjects during free living or scripted activity experiments. Then, the quality of the performed activities such as meal preparation or cleaning, could be assessed. Also, the relationship between PD symptoms like bradykinesia, and the performed activities could be modelled

Behaviour analysis of the PD patients **–** ML algorithms could be applied to model the behavioural patterns of PD vs non-PD subjects. Such models could be also trained to distinguish between the behaviour of the PD patients during 'on' and 'off' medication periods. Moreover, the patterns in the sleeping habits, mobility, and urinary function of the patients could be learned and analysed.

Modelling the progression of PD temporally **–** The above-mentioned predictions could be monitored through time by modelling the progression of the disease via ML algorithms that provide temporal analysis of the input data. Specifically, models like HMMs or Recurrent Neural Networks (RNNs), which can produce predictions per time point, could be trained to temporally model the PD symptoms. Therefore, for example, PD symptoms could be assessed at different times of the day during the study.

Analysing the information collected from screening and diaries **–** The questionnaires and tests performed during screening (Contact B), as well as the diaries filled by the patients during their stay in the SPHERE house, would provide information which could be summarised via descriptive



For peer review only - http://bmjopen.bmj.com/site/about/guidelines.xhtml

UOB Confidential

statistics. Additionally, associations with the data collected from various sensors, for example, environmental sensors, could be modelled via ML.

Evaluating traditional vs state-of-the-art ML algorithms **–** As presented in (34), there are many ML algorithms which can be applied to model PD. We would have the ability to assess the performance of such alternative algorithms. Specifically, a comparison between classical ML algorithms, such as SVMs or RFs, against state-of the-art deep learning-based approaches for modelling PD is of interest, as the latter has achieved high performance in areas such as computer vision.

Evaluating single vs multi-modal approaches **–** Due to the multi-sensor data collection platform within the SPHERE house, the performance of ML models which are based on single or multiple modalities could be assessed. In a multi-modal approach, methods for fusing data from different modalities, e.g. early or late fusion methods, could be also evaluated to determine which modalities are most effective.

Qualitative data collection and analysis

A semi-structured interview will be conducted with each participant at the end of the study exploring their experience of the SPHERE technology, with a focus on the tolerability and acceptability of such technology for people with PD and those close to them. An interview topic guide is shown in Box 1. Interview transcripts will be read, re-read, and the data gathered will be analysed thematically, using an iterative-inductive approach (35), with methods drawn from Braun and Clarke (36).

Box 1. Interview Topic Guide

- Background experience with technology
- Experience of the SPHERE sensor platform
- Thoughts about the sensor data collected
- The study information received relating to the SPHERE technology
- Questions about the study experience specific to PD and those close to people with PD

Additionally, two questionnaires will be used to augment the qualitative data produced from the interviews. The first questionnaire will be a standardised tool evaluating the participants' attitudes towards and experience of technology: using four attitude-based subscales (looking at positive attitudes, negative attitudes, technological anxiety/dependence, and attitudes towards task-switching) from The Media and Technology Usage and Attitudes Scale (37). This will allow us to interpret the interview responses in the context of a participant's prior background exposure to technology. The second questionnaire comprises three short questions exploring feedback about the sensors or study in general regarding thoughts that could have occurred to the participant in the days after they left the SPHERE house. These questions are listed in Box 2. This enables us to ensure we understand the experience of this sensor platform as fully as possible.



For peer review only - http://bmjopen.bmj.com/site/about/guidelines.xhtml

UOB Confidential

Box 2. Questions following-up the SPHERE house stay (Contact E questionnaire)

1. Following the PD SENSORS study, now that you have finished the study and been at home for a while, do you have any further thoughts to feedback to us about the SPHERE sensors that you lived with for 5 days?
2. Specifically, in relation to your PD/supporting role for a person with PD, was there anything further you wanted us to know about or understand?
3. Is there anything else at all that you felt important to mention to us regarding your study experience?

Data management and security

The day-to-day management of the study including data management will be coordinated by the Principal Investigator of the study. All the data collected will be fully anonymised or pseudonymised where possible. Unique study identification numbers will be assigned to each participant. Access to data will be limited to SPHERE researchers and collaborators only, who must be registered (to use the data) with managed access and permissions. Directly identifiable data (e.g. name, address, telephone number) will be held securely and separately from other study data. Data procedures will be in accordance with the General Data Protection Regulation. If a participant withdraws, the data that is already obtained will be kept by the study team. Under a Service Level Agreement between the University of Bristol and University Hospitals Bristol NHS Foundation Trust, the Trust will monitor 10% of the University's Sponsored studies. In line with the National Institute for Health and Research guidance which encourages sharing anonymised datasets, we will seek informed consent from participants for their anonymous data to be shared with other researchers. The data management will be reviewed on a regular basis by an in-house Data Monitoring Committee consisting of data experts, engineers and movement disorders clinicians.

Data Statement

SPHERE intends to make any research data produced as widely and freely available as possible in a timely and responsible manner. The information would be suitable for sharing as we are developing novel and potentially useful ways to assess PD. However, some portions of research data will not be suitable for sharing, e.g., directly identifiable information such as names and addresses. We will seek consent from participants for pseudonymised subsets of their data to be shared with other researchers. This is in anticipation that data sharing and access to such datasets may become mandatory in the coming years.

Data published by the University of Bristol (UoB) Repository can have open or controlled data access. Published datasets will appear in a standardised, online 'data catalogue' accompanied by its deposit record. These deposits are also made visible to search engines such as Google, to help maximise their visibility and impact. We will also create records of digital and/or non-digital data produced in Pure (http://www.bristol.ac.uk/red/research-policy/pure/).

**Further Study Detail**

Collaboration with IXICO plc



For peer review only - http://bmjopen.bmj.com/site/about/guidelines.xhtml

UOB Confidential

The University of Bristol and IXICO plc will collaborate on specific outcomes, as well as through the sharing of data and results, the loan of the wearable biosensors (Axivity AX3 and GENEActiv Original) and gait mat (Zeno walkway) and in-kind contributions of scientific expertise and study operations. The topics explored within this collaboration will include the feasibility of labelling total body bradykinesia from colour video, to developed automated detection of bradykinesia from biosensor data, biosensor device comparability, and relationships between sensor-based measurements from the gait mat and wearable devices and between sensor-based measurements and traditional clinical measures of motor symptoms, in addition to ADL detection from wearable sensor data.

Safety reporting

Any adverse event (AE) will be assessed and acted upon by the research team. All AEs will be recorded in the study or project file with a note that will identify when the event occurred, the details of the AE, any potential study relation, action taken and resolution / closure of the AE. An assessment of seriousness will be made by the researcher and serious adverse events (SAEs) will be reported in line with current legislation and university guidance.

In addition, all SAEs will be reported to the NHS Research Ethics Committee in the Annual Progress Report.

Amendments

Any amendments to protocol design will be communicated to relevant parties including North Bristol NHS Trust and the Research Ethics Committee.

Patient and public involvement

The Bristol Movement Disorders Health Integration Team (MOVE-HIT) has an active patient and public involvement (PPI) group which includes people with Parkinson's disease and their partners/carers. The MOVE-HIT PPI group meets regularly and has been involved in providing extensive advice around the study design, as well as the development of the participant information sheets, consent forms and participant diaries. The opinions of members of this PPI group on study design have been collected both in face-to-face group and individual consultations and in a survey to wider group members. We plan further ongoing consultation, both during the middle of the study to discuss the group's views on study progress and at the end of the study to consult on the plain English report on the study findings and to identify dissemination outlets.

The PD SENSORS research team are also collaborating with Lyndsey Isaacs, a Trustee of The Cure Parkinson's Trust (CPT), who facilitated the presentation of a PD SENSORS study design survey to the delegates at a CPT Research Update Meeting. This survey was also made more widely available on the CPT website. The research team will have ongoing liaison with CPT representatives during the study to discuss aspects of study management such as dissemination of research findings. The CPT have agreed to be a forum through which to disseminate the study Poster to allow interested persons to express interest in participating in PD SENSORS.

Ethics and Dissemination



For peer review only - http://bmjopen.bmj.com/site/about/guidelines.xhtml

UOB Confidential

The SPHERE house has been safety checked by an experienced occupational therapist practitioner with an interest in PD and relevant adaptations made.

It is anticipated that the scientific results of the study and its various elements will be published both via presentation at national or international conferences and through publication in peer-reviewed scientific journals/publications.

Funding

This work was performed under the SPHERE Next Steps Project funded by the UK Engineering and Physical Sciences Research Council (EPSRC), Grant EP/R005273/1.

This work is supported by the Elizabeth Blackwell Institute for Health Research, University of Bristol, and the Wellcome Trust Institutional Strategic Support Fund, grant code: 204813/Z/16/Z, by The Cure Parkinson's Trust, grant code AW021 and by IXICO plc, grant code R101507-101.

Dr Jonathan de Pass and Mrs Georgina de Pass made a charitable donation to the University of Bristol (of which Jonathan is a graduate) through the Development and Alumni Relations Office to support research into Parkinson's Disease. The funding pays for the salary of CM, the PhD student and Clinical Research Fellow, and she reports to the donors on her progress, but they do not have any influence on the direction of the study.

Author Statement

CM drafted the study protocol, worked with all contributors to improve and finalise details and continues as the study Principal Investigator. IC and AW supervise the studies and work of CM; AW is study Chief Investigator; IC is Principal of SPHERE-IRC; both have major influence on the study's direction. ET has advised on data management. KK and RoM have done pilot work and have advised on study direction. MM, FH, RyM, ET and SW have been involved in the computer science and analytics planning. JC, AH, MR and RE have given practical input on study tools and documentation. OW and HM are representatives of the Public and Patient Involvement groups which have had strong influence on study design. LR and WM have added expertise in the field to advise CM on early study design.

The study sponsor is the University of Bristol (research-governance@bristol.ac.uk). The sponsor has had no role in study design, collection, management, analysis, interpretation of the data, writing the report or the decision to submit the study for publications and will have no ultimate authority over these activities.

Conflicts of interests

K Kinnunen is employed by IXICO plc, who have provided some financial support of the study. The research may lead to the development of products which may be licensed to IXICO plc.

None of the other authors have any financial or other competing interests to declare.

References

1. Jankovic J. Parkinson's disease: clinical features and diagnosis. *J Neurol Neurosurg Psychiatry* 2008;79(4):368–76.



For peer review only - http://bmjopen.bmj.com/site/about/guidelines.xhtml

UOB Confidential

2. Fearnley JM, Lees AJ. Ageing and Parkinson's disease: substantia nigra regional selectivity. *Brain* 1991;114(Pt 5):2283–301.
3. Lang AE, Melamed E, Poewe W, et al. Trial designs used to study neuroprotective therapy in Parkinson's disease. *Mov Disord* 2013;28(1):86–95.
4. Goetz CG, Fahn S, Martinez-Martin P, et al. Movement Disorder Society-sponsored revision of the Unified Parkinson's Disease Rating Scale (MDS-UPDRS): Process, format, and clinimetric testing plan. *Mov Disord* 2007;22(1):41–7.
5. Goetz CG, Tilley BC, Shaftman SR, et al. Movement Disorder Society-Sponsored Revision of the Unified Parkinson's Disease Rating Scale (MDS-UPDRS): Scale presentation and clinimetric testing results. *Mov Disord* 2008;23(15):2129–70.
6. Post B, Merkus MP, de Bie RMA, et al. Unified Parkinson's disease rating scale motor examination: are ratings of nurses, residents in neurology, and movement disorders specialists interchangeable? *Mov Disord* 2005 Dec;20(12):1577–84.
7. Mcghee DJM, Ritchie CW, Zajicek JP, et al. A review of clinical trial designs used to detect a disease-modifying effect of drug therapy in Alzheimer's disease and Parkinson's disease. *BMC Neurol* 2016;16:92.
8. Robles-García V, Corral-Bergantiños Y, Espinosa N, et al. Spatiotemporal Gait Patterns During Overt and Covert Evaluation in Patients With Parkinson´s Disease and Healthy Subjects: Is There a Hawthorne Effect? *J Appl Biomech* 2015;31(3):189–94.
9. Cubo E, Mariscal N, Solano B, Becerra V, Armesto D, Calvo S, et al. Prospective study on cost-effectiveness of home-based motor assessment in Parkinson's disease. *J Telemed Telecare* 2017;23(2):328–38.
10. Duncan RP, Earhart GM. Measuring participation in individuals with Parkinson disease: Relationships with disease severity, quality of life, and mobility. *Disabil Rehabil* 2011;33(15–16):1440–6.
11. Silva de Lima AL, Smits T, Darweesh SKL, et al. Home-based monitoring of falls using wearable sensors in Parkinson's disease. *Mov Disord* 2019;35:109–15.
12. Van Uem JMT, Isaacs T, Lewin A, et al. A viewpoint on wearable technology-enabled measurement of wellbeing and health-related quality of life in Parkinson's disease. *Journal of Parkinson's Disease* 2016;6(2):279–87.
13. Godinho C, Domingos J, Cunha G, et al. A systematic review of the characteristics and validity of monitoring technologies to assess Parkinson's disease. *J Neuroeng Rehabil* 2016;13:24.
14. Morgan C, Rolinski M, McNaney R, et al. Systematic Review Looking at the Use of Technology to Measure Free-Living Symptom and Activity Outcomes in Parkinson's Disease in the Home or Home-like environment. *Journal of Parkinson's Disease* 2020;10:429-454.
15. Griffiths RI, Kotschet K, Arfon S, et al. Automated assessment of bradykinesia and dyskinesia in Parkinson's disease. *J Parkinsons Dis* 2012;2(1):47–55.
16. Dranca L, de Abetxuko Ruiz de Mendarozketa L, Goñi A, et al. Using Kinect to classify Parkinson's disease stages related to severity of gait impairment. *BMC Bioinformatics* 2018;19(1).
17. Artusi, C.A., Imbalzano, G., Sturchio, A. *et al.* Implementation of Mobile Health Technologies in Clinical Trials of Movement Disorders: Underutilized Potential. *Neurotherapeutics* (2020). https://doi.org/10.1007/s13311-020-00901-x
18. Espay AJ, Hausdorff JM, Sánchez-Ferro Á, et al. A roadmap for implementation of patient-centered digital outcome measures in Parkinson's disease obtained using mobile health technologies. *Mov Disord* 2019;34(5):657–63.
19. Ellis T, Cavanaugh JT, Earhart GM, et al. Which Measures of Physical Function and Motor Impairment Best Predict Quality of Life in Parkinson's Disease? *Parkinsonism Relat Disord* 2011;17(9):693-7.
20. Del Din S, Godfrey A, Mazzà C, et al. Free-living monitoring of Parkinson's disease: Lessons from the field. *Mov Disord* 2016;31(9):1293–313.



For peer review only - http://bmjopen.bmj.com/site/about/guidelines.xhtml

UOB Confidential

21. Woznowski P, Burrows A, Diethe T, et al. SPHERE: A Sensor Platform for Healthcare in a Residential Environment. Designing, Developing, and Facilitating Smart Cities. Cham: Springer International Publishing 2017:315–33.
22. Zhu N, Diethe T, Camplani M, et al. Bridging e-Health and the Internet of Things: The SPHERE Project. *IEEE Intell Syst* 2015 Jul;30(4):39–46. Available from: http://ieeexplore.ieee.org/document/7156004/
23. Burrows A, Gooberman-Hill R, Coyle D. Empirically derived user attributes for the design of home healthcare technologies. *Pers Ubiquitous Comput* 2015;19(8):1233–45.
24. Burrows A, Gooberman-Hill R, Coyle D. Shared Language and the Design of Home Healthcare Technology. *Proceedings of the CHI Conference on Human Factors in Computing Systems* 2016:3584-94.
25. Grant S, Blom AW, Whitehouse MR, et al. Using home sensing technology to assess outcome and recovery after hip and knee replacement in the UK: The HEmiSPHERE study protocol. *BMJ Open* 2018;8(7):e021862.
26. Camplani M, Hannuna S, Mirmehdi M, et al. Real-time RGB-D Tracking with Depth Scaling Kernelised Correlation Filters and Occlusion Handling. *Procedings of the British Machine Vision Conference* 2015:145.1-145.11.
27. Tao L, Burghardt T, Hannuna S, et al. A comparative home activity monitoring study using visual and inertial sensors. *17th International Conference on E-health Networking, Application & Services (HealthCom)* 2015:644–7.
28. Hughes AJ, Daniel SE, Kilford L, et al. Accuracy of clinical diagnosis of idiopathic Parkinson's disease: A clinico-pathological study of 100 cases. *J Neurol Neurosurg Psychiatry* 1992;55(3):181–4.
29. Trenkwalder C, Kohnen R, Högl B, et al. Parkinson's disease sleep scale-validation of the revised version PDSS-2. *Mov Disord* 2011 Mar;26(4):644–52.
30. Martinez-Martin P, Rodriguez-Blazquez C, Abe K, et al. International study on the psychometric attributes of the Non-Motor Symptoms Scale in Parkinson disease. *Neurology* 2009;73(19):1584–91.
31. Morris S, Morris ME, Iansek R. Reliability of measurements obtained with the Timed "Up & Go" test in people with Parkinson disease. *Phys Ther* 2001 Feb;81(2):810–8.
32. Stiasny-Kolster K, Mayer G, Schäfer S, et al. The REM sleep behavior disorder screening questionnaire-A new diagnostic instrument. *Mov Disord* 2007;22(16):2386–93.
33. Marinus J, Ramaker C, van Hilten JJ, et al. Health related quality of life in Parkinson's disease: a systematic review of disease specific instruments. *J Neurol Neurosurg Psychiatry* 2002;72(2):241–8.
34. Belić M, Bobić V, Badža M, et al. Artificial intelligence for assisting diagnostics and assessment of Parkinson's disease—A review. *Clinical Neurology and Neurosurgery* 2019;184:105442.
35. O'Reilly K. Ethnographic methods [second edition]. Ethnographic Methods. London: Routledge; 2012.
36. Braun V, Clarke V. Using thematic analysis in psychology. *Qual Res Psychol*. 2006;3(2):77–101.
37. Rosen LD, Whaling K, Carrier LM, Cheever NA, Rokkum J. The Media and Technology Usage and Attitudes Scale: An empirical investigation. *Comput Human Behav*. 2013;29(6):2501–11.

Figure legends

Figure 1. Flow chart detailing outline of PD SENSORS study contacts.

*PIS = Participant Information Sheet*

Figure 2. A visualisation of the day-to-day participant activities during Contact D, the SPHERE house stay.



For peer review only - http://bmjopen.bmj.com/site/about/guidelines.xhtml

UOB Confidential

*OFF medications = the participant with PD in practically-defined 'off' medication state which entails withholding dopaminergic medications for a period of more than 12 hours; ON medications = the participant with PD having taken their dopaminergic medications.*

For peer review only



For peer review only - http://bmjopen.bmj.com/site/about/guidelines.xhtml

UOB Confidential

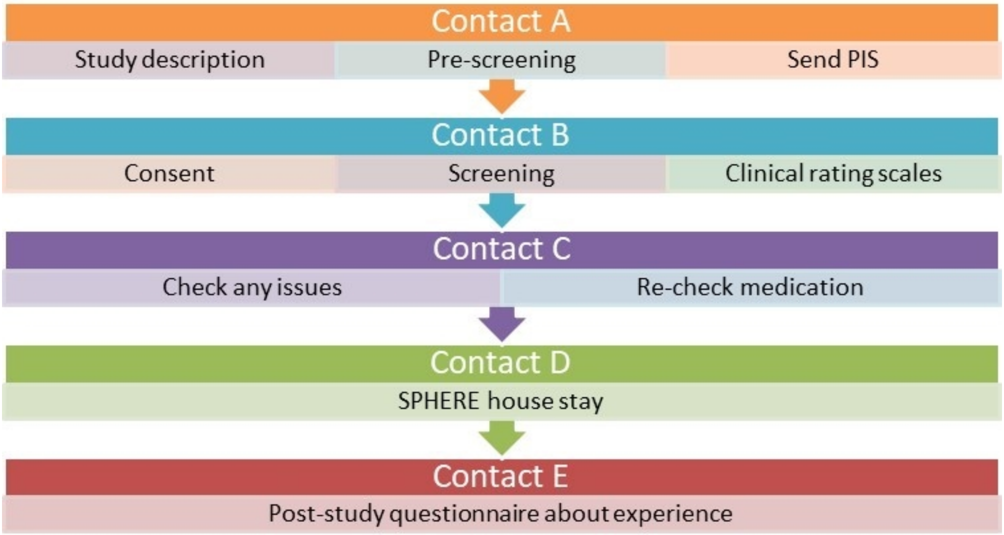


Figure 1. Flow chart detailing outline of PD SENSORS study contacts.

PIS = Participant Information Sheet

159x89mm (120 x 120 DPI)

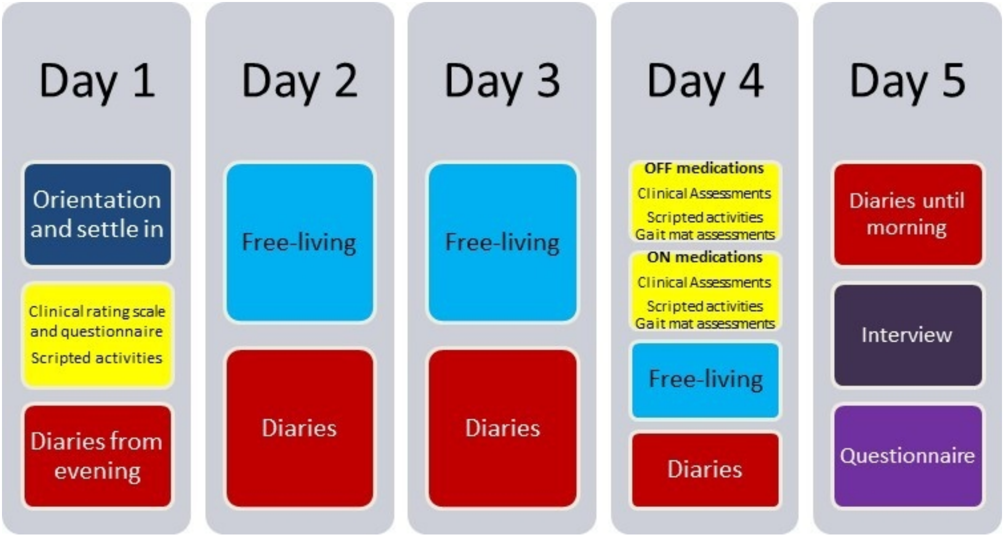


Figure 2. A visualisation of the day-to-day participant activities during Contact D, the SPHERE house stay.

OFF medications = the participant with PD in practically-defined 'off' medication state which entails withholding dopaminergic medications for a period of more than 12 hours; ON medications = the participant with PD having taken their dopaminergic medications.

159x84mm (120 x 120 DPI)

**Supplementary Material**

A list of some of the specific movements/actions to be evaluated in the scripted activities during Contact D

- Spreading hummus on bread
- Cutting
- Pouring
- Scooping
- Stirring
- Turning tap on and off
- Wiping surface
- Dispensing soap

For peer review only

# Reporting checklist for protocol of a clinical trial.

Based on the SPIRIT guidelines.

## Instructions to authors

Complete this checklist by entering the page numbers from your manuscript where readers will find each of the items listed below.

Your article may not currently address all the items on the checklist. Please modify your text to include the missing information. If you are certain that an item does not apply, please write "n/a" and provide a short explanation.

Upload your completed checklist as an extra file when you submit to a journal.

In your methods section, say that you used the SPIRITreporting guidelines, and cite them as:

Chan A-W, Tetzlaff JM, Altman DG, Laupacis A, Gøtzsche PC, Krleža-Jerić K, Hróbjartsson A, Mann H, Dickersin K, Berlin J, Doré C, Parulekar W, Summerskill W, Groves T, Schulz K, Sox H, Rockhold FW, Rennie D, Moher D. SPIRIT 2013 Statement: Defining standard protocol items for clinical trials. Ann Intern Med. 2013;158(3):200-207

| | | Reporting Item | Page Number |
|---|---|---|---|
| **Administrative information** | | | |
| Title | #1 | Descriptive title identifying the study design, population, interventions, and, if applicable, trial acronym | 1 |

For peer review only

| | | | |
|---|---|---|---|
| Trial registration | #2a | Trial identifier and registry name. If not yet registered, name of intended registry | 1 |
| Trial registration: data set | #2b | All items from the World Health Organization Trial Registration Data Set | N/A |
| Protocol version | #3 | Date and version identifier | 4 |
| Funding | #4 | Sources and types of financial, material, and other support | 14 |
| Roles and responsibilities: contributorship | #5a | Names, affiliations, and roles of protocol contributors | 14-15 |
| Roles and responsibilities: sponsor contact information | #5b | Name and contact information for the trial sponsor | 15 |
| Roles and responsibilities: sponsor and funder | #5c | Role of study sponsor and funders, if any, in study design; collection, management, analysis, and interpretation of data; writing of the report; and the decision to submit the report for publication, including whether they will have ultimate authority over any of these activities | 15 |
| Roles and responsibilities: committees | #5d | Composition, roles, and responsibilities of the coordinating centre, steering committee, endpoint adjudication committee, data management team, and other individuals or groups overseeing the trial, if applicable (see Item 21a for data | 13, 15 |

For peer review only

| | | | |
|---|---|---|---|
| | | monitoring committee) | |
| **Introduction** | | | |
| Background and rationale | #6a | Description of research question and justification for undertaking the trial, including summary of relevant studies (published and unpublished) examining benefits and harms for each intervention | 3-5 |
| Background and rationale: choice of comparators | #6b | Explanation for choice of comparators | 11 |
| Objectives | #7 | Specific objectives or hypotheses | 5 |
| Trial design | #8 | Description of trial design including type of trial (eg, parallel group, crossover, factorial, single group), allocation ratio, and framework (eg, superiority, equivalence, non-inferiority, exploratory) | 5 |
| **Methods: Participants, interventions, and outcomes** | | | |
| Study setting | #9 | Description of study settings (eg, community clinic, academic hospital) and list of countries where data will be collected. Reference to where list of study sites can be obtained | 5 |
| Eligibility criteria | #10 | Inclusion and exclusion criteria for participants. If applicable, | 6 |

For peer review only

| | | | |
|---|---|---|---|
| | | eligibility criteria for study centres and individuals who will perform the interventions (eg, surgeons, psychotherapists) | |
| Interventions: description | #11a | Interventions for each group with sufficient detail to allow replication, including how and when they will be administered | 6-9 |
| Interventions: modifications | #11b | Criteria for discontinuing or modifying allocated interventions for a given trial participant (eg, drug dose change in response to harms, participant request, or improving / worsening disease) | N/A |
| Interventions: adherance | #11c | Strategies to improve adherence to intervention protocols, and any procedures for monitoring adherence (eg, drug tablet return; laboratory tests) | N/A |
| Interventions: concomitant care | #11d | Relevant concomitant care and interventions that are permitted or prohibited during the trial | 6 |
| Outcomes | #12 | Primary, secondary, and other outcomes, including the specific measurement variable (eg, systolic blood pressure), analysis metric (eg, change from baseline, final value, time to event), method of aggregation (eg, median, proportion), and time point for each outcome. Explanation of the clinical relevance of chosen efficacy and harm outcomes is strongly recommended | 5, 7, 11-13 |
| Participant timeline | #13 | Time schedule of enrolment, interventions (including any run-ins and washouts), assessments, and visits for participants. A schematic diagram is highly recommended (see Figure) | 7 |

For peer review only

| | | | |
|---|---|---|---|
| Sample size | #14 | Estimated number of participants needed to achieve study objectives and how it was determined, including clinical and statistical assumptions supporting any sample size calculations | 11 |
| Recruitment | #15 | Strategies for achieving adequate participant enrolment to reach target sample size | 6 |
| **Methods: Assignment of interventions (for controlled trials)** | | | |
| Allocation: sequence generation | #16a | Method of generating the allocation sequence (eg, computer-generated random numbers), and list of any factors for stratification. To reduce predictability of a random sequence, details of any planned restriction (eg, blocking) should be provided in a separate document that is unavailable to those who enrol participants or assign interventions | N/A |
| Allocation concealment mechanism | #16b | Mechanism of implementing the allocation sequence (eg, central telephone; sequentially numbered, opaque, sealed envelopes), describing any steps to conceal the sequence until interventions are assigned | N/A |
| Allocation: implementation | #16c | Who will generate the allocation sequence, who will enrol participants, and who will assign participants to interventions | N/A |
| Blinding | #17a | Who will be blinded after assignment to interventions (eg, trial | N/A |

For peer review only

| | | | |
|---|---|---|---|
| (masking) | | participants, care providers, outcome assessors, data analysts), and how | |
| Blinding (masking): emergency unblinding | #17b | If blinded, circumstances under which unblinding is permissible, and procedure for revealing a participant's allocated intervention during the trial | N/A |
| **Methods: Data collection, management, and analysis** | | | |
| Data collection plan | #18a | Plans for assessment and collection of outcome, baseline, and other trial data, including any related processes to promote data quality (eg, duplicate measurements, training of assessors) and a description of study instruments (eg, questionnaires, laboratory tests) along with their reliability and validity, if known. Reference to where data collection forms can be found, if not in the protocol | 7-10 |
| Data collection plan: retention | #18b | Plans to promote participant retention and complete follow-up, including list of any outcome data to be collected for participants who discontinue or deviate from intervention protocols | 13 |
| Data management | #19 | Plans for data entry, coding, security, and storage, including any related processes to promote data quality (eg, double data | 13 |

For peer review only

| | | | |
|---|---|---|---|
| | | entry; range checks for data values). Reference to where details of data management procedures can be found, if not in the protocol | |
| Statistics: outcomes | #20a | Statistical methods for analysing primary and secondary outcomes. Reference to where other details of the statistical analysis plan can be found, if not in the protocol | 11-13 |
| Statistics: additional analyses | #20b | Methods for any additional analyses (eg, subgroup and adjusted analyses) | 11-13 |
| Statistics: analysis population and missing data | #20c | Definition of analysis population relating to protocol non-adherence (eg, as randomised analysis), and any statistical methods to handle missing data (eg, multiple imputation) | N/A |
| **Methods: Monitoring** | | | |
| Data monitoring: formal committee | #21a | Composition of data monitoring committee (DMC); summary of its role and reporting structure; statement of whether it is independent from the sponsor and competing interests; and reference to where further details about its charter can be found, if not in the protocol. Alternatively, an explanation of why a DMC is not needed | 12 |
| Data monitoring: interim analysis | #21b | Description of any interim analyses and stopping guidelines, including who will have access to these interim results and | N/A |

For peer review only

| | | | |
|---|---|---|---|
| | | make the final decision to terminate the trial | |
| Harms | #22 | Plans for collecting, assessing, reporting, and managing solicited and spontaneously reported adverse events and other unintended effects of trial interventions or trial conduct | 9-10 |
| Auditing | #23 | Frequency and procedures for auditing trial conduct, if any, and whether the process will be independent from investigators and the sponsor | 13 |
| **Ethics and dissemination** | | | |
| Research ethics approval | #24 | Plans for seeking research ethics committee / institutional review board (REC / IRB) approval | 6 |
| Protocol amendments | #25 | Plans for communicating important protocol modifications (eg, changes to eligibility criteria, outcomes, analyses) to relevant parties (eg, investigators, REC / IRBs, trial participants, trial registries, journals, regulators) | 11 |
| Consent or assent | #26a | Who will obtain informed consent or assent from potential trial participants or authorised surrogates, and how (see Item 32) | 8 |
| Consent or assent: ancillary studies | #26b | Additional consent provisions for collection and use of participant data and biological specimens in ancillary studies, if applicable | N/A |
| Confidentiality | #27 | How personal information about potential and enrolled participants will be collected, shared, and maintained in order | 14 |

For peer review only

| | | | |
|---|---|---|---|
| | | to protect confidentiality before, during, and after the trial | |
| Declaration of interests | #28 | Financial and other competing interests for principal investigators for the overall trial and each study site | 14 |
| Data access | #29 | Statement of who will have access to the final trial dataset, and disclosure of contractual agreements that limit such access for investigators | 13 |
| Ancillary and post trial care | #30 | Provisions, if any, for ancillary and post-trial care, and for compensation to those who suffer harm from trial participation | N/A |
| Dissemination policy: trial results | #31a | Plans for investigators and sponsor to communicate trial results to participants, healthcare professionals, the public, and other relevant groups (eg, via publication, reporting in results databases, or other data sharing arrangements), including any publication restrictions | 13-14 |
| Dissemination policy: authorship | #31b | Authorship eligibility guidelines and any intended use of professional writers | N/A |
| Dissemination policy: reproducible research | #31c | Plans, if any, for granting public access to the full protocol, participant-level dataset, and statistical code | 13 |

**Appendices**

| | | | |
|---|---|---|---|
| Informed consent materials | #32 | Model consent form and other related documentation given to participants and authorised surrogates | Appendix |

For peer review only

| | | | |
|---|---|---|---|
| Biological specimens | #33 | Plans for collection, laboratory evaluation, and storage of biological specimens for genetic or molecular analysis in the current trial and for future use in ancillary studies, if applicable | N/A |

None The SPIRIT checklist is distributed under the terms of the Creative Commons Attribution License CC-BY-ND 3.0. This checklist can be completed online using https://www.goodreports.org/, a tool made by the EQUATOR Network in collaboration with Penelope.ai

For peer review only
